# Influence of the Acid Reactivity of Carbenium Ions in Zeolites on the Methanol‐to‐Olefins Process

**DOI:** 10.1002/anie.202514759

**Published:** 2025-10-06

**Authors:** Luca Maggiulli, Vitaly L. Sushkevich, Annika E. Enss, Felix Studt, Jeroen A. van Bokhoven, Davide Ferri

**Affiliations:** ^1^ PSI Center for Energy and Environmental Sciences Paul Scherrer Institute Villigen PSI CH‐5232 Switzerland; ^2^ Institute for Chemical and Bioengineering ETH Zurich Vladimir‐Prelog‐Weg 1 Zurich CH‐8093 Switzerland; ^3^ Institute for Catalysis Research and Technology Karlsruhe Institute of Technology Hermann‐von‐Helmholtz‐Platz 1 D‐76344 Eggenstein‐Leopoldshafen Germany; ^4^ Institute for Chemical Technology and Polymer Chemistry Karlsruhe Institute of Technology D‐76131 Karlsruhe Germany

**Keywords:** Adsorption energy, Brønsted acid, Carbenium ion, DFT, In situ FTIR, Zeolites

## Abstract

Carbocations are typical intermediates in acid catalyzed reactions in organic chemistry synthesis. Confinement within the framework of zeotype materials distinguishes the function of carbenium ions as catalytic centers in various chemical processes. A selective and reversible deprotonation event of benzenium ions built in H‐ZSM‐5 during methanol conversion was observed by experiment and theory as a result of CD_3_CN dosage, denoting their higher acid reactivity compared to the cyclopentenyl cations, which undergo deprotonation only in presence of pyridine. This study uncovers the inverse relationship between the acid reactivity of carbenium ions of different nature and their stability in the surface‐bound state.

## Graphical abstract

Herein, we studied the Brønsted acid reactivity of polymethylbenzenium and alkyl‐substituted cyclopentenyl cations, key intermediates in the methanol‐to‐olefins process in ZSM‐5 zeolite. CD_3_CN selectively and reversibly deprotonates benzenium ions, while cyclopentenyl cations require stronger bases. These results reveal a reactivity scale and provide insight into carbocation chemistry in reactive environments.

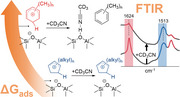

Carbocations play a vital role as key reactive intermediates in modern industrial synthetic organic chemistry^[^ ^1^ ^]^ and are central to organic chemistry teaching and training.^[^ ^2^, ^3^ ^]^ Their electrophilic nature makes them unstable and highly reactive toward various organic transformations, e.g. nucleophilic attacks, rearrangements, and elimination reactions, through which they achieve a more stable configuration. Carbenium ions are a class of carbocations with a positively charged trivalent carbon atom and a (close to) planar sp^2^ hybridization^[^ ^4^ ^]^ whose structure has driven research interest for over a century. In the methanol‐to‐olefins (MTO) process, olefins formation occurs through a hydrocarbon pool catalysis, where carbenium ions act as active centers for the conversion of methanol into olefins together with olefinic and aromatic hydrocarbons.^[^ ^5^ ^]^ Various carbenium ions have been identified in zeolites by spectroscopy during the MTO process,^[^ ^6^, ^7^, ^8^, ^9^ ^]^ whose structure is analogous to that of the same ions generated in the liquid phase.^[^ ^10^ ^]^ At typical reaction conditions, carbenium ions are generated by protonation of an unsaturated hydrocarbon or by hydride transfer and are then stabilized by interaction with the zeolite framework. In particular, the alkyl‐substituted cyclopentenyl cations (CP^+^) and polymethylbenzenium ions (PMB^+^) (Scheme 1) are the most discussed cyclic ions in the reaction mechanism and are considered to be mechanistically related.^[^ ^8^, ^11^, ^12^, ^13^ ^]^ CP^+^ was proposed to be an early‐stage species that can interconvert into PMB^+^.^[^ ^14^, ^15^ ^]^

**Scheme 1:**
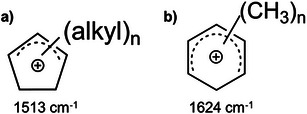
Structure of carbenium ions confined within H‐ZSM‐5: a) alkyl‐substituted cyclopentenyl cation and b) polymethylbenzenium ion with their characteristic ν(C─C═C)^+^ vibrational mode.^[^ ^8^, ^9^ ^].^

While theory and experimental evidence can explain the formation of the first C─C bond from methanol, a substantial lack of understanding remains on the nature of the molecular steps involving the carbenium ions responsible for the transformations into products. Three scenarios are anticipated for their interaction with a base such as methanol:^[^ ^16^ ^]^ 1) no reaction beyond hydrogen bonding, 2) deprotonation of the carbenium ion to form a hydrogen bonded complex, or 3) formation of an onium complex as a result of Lewis acid–base reaction with the carbenium ion acting as Lewis acid. While the 1,3‐dimethylcyclopentenyl cation does not form onium ions with water, dimethyl ether, acetone or carbon monoxide, deprotonation or formation of onium ions occurs only with strong bases (ammonia, phosphine, pyridine, trimethylphospine).^[^ ^16^ ^]^ On the other hand, quantum chemical calculations predict that the plausible proton transfer reaction from PMB^+^ to a methanol molecule and the subsequent methylation of the arene ring possess low energy barriers.^[^ ^17^ ^]^ It is therefore desirable to understand the possible pathways of carbenium ions interaction with bases and to clarify their potential different reactivity, which can lead to different product selectivities in the MTO process. In the present work, we observe by experiment and theory the selective and reversible deprotonation of PMB^+^ confined within H‐ZSM‐5 upon interaction with CD_3_CN as weak base.

Two H‐ZSM‐5 zeolites were obtained from different activation protocols of the ammonium form of the same parent material (Section 1.1 of the Supplementary Information; Figures S1–S6, Tables S1 and S2). Z5 exhibited a higher ratio of Brønsted acid sites to Lewis acid sites (BAS/LAS ratio, 9.08) than Z5_EFAL (1.29) in agreement with the observation of extra‐framework aluminum in the FTIR spectrum of Z5_EFAL (EFAL; ν(OH) = 3665 cm^−1^; Figure S6).

After exposure of Z5_EFAL to methanol vapors at 543 K and evacuation, the signals of the ν(C─C═C)^+^ mode of CP^+^ (1513 cm^−1^) and PMB^+^ (1624 cm^−1^) appeared in its transmission FTIR spectra (Figure 1a–c; Section 1.2 of the Supplementary Information).

**Figure 1:**
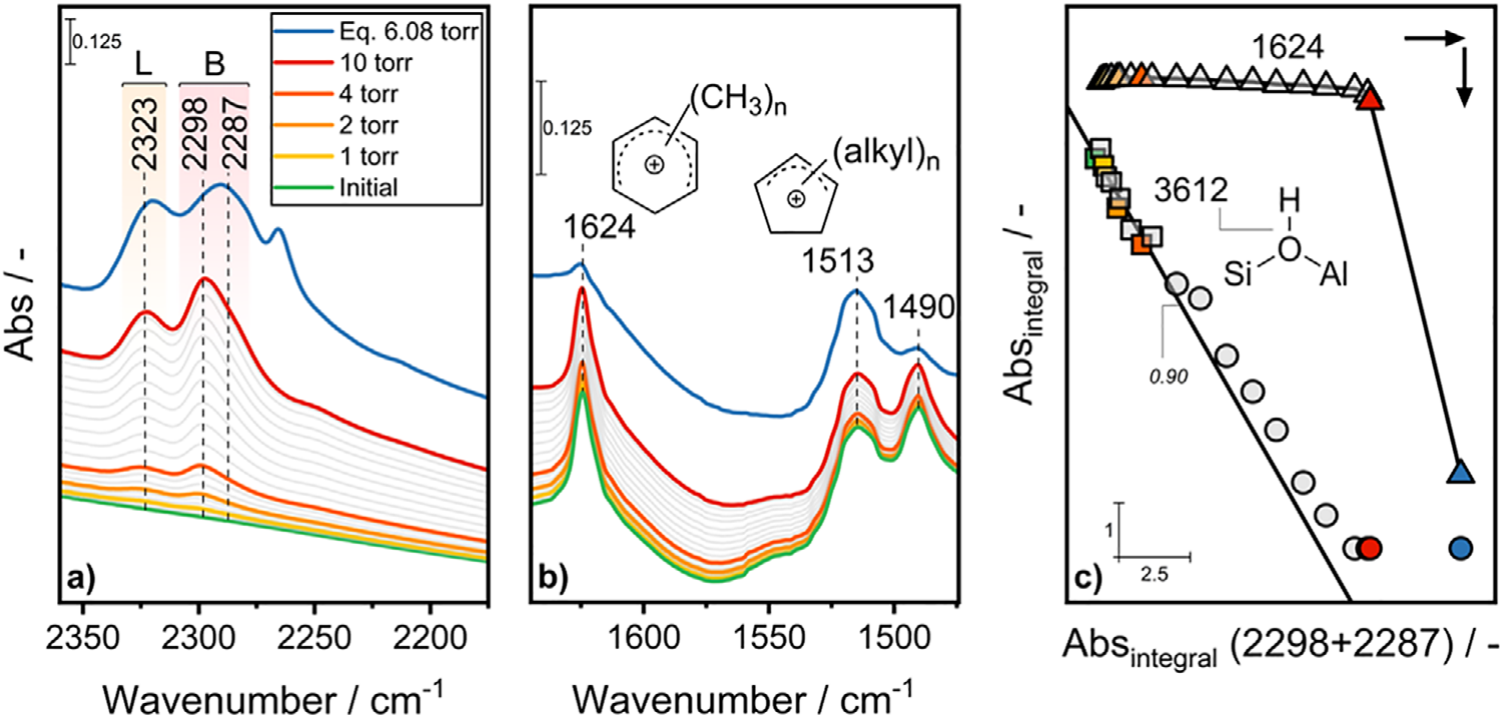
In situ FTIR spectra of methanol‐treated Z5_EFAL in the a) ν(C≡N) and b) ν(C═C) regions during CD_3_CN titration at room temperature. “L” and “B” stand for LAS and BAS complexed with CD_3_CN, respectively. c) Correlation between the areas of the bands of the ν(O─H) of BAS (3612 cm^−1^) and of the ν(C─C═C)^+^ mode of PMB+ (1624 cm^−1^) with the areas of the bands of the ν(C≡N) mode of the BAS–NCCD_3_ adduct (Abs_integral_(2298 + 2287)). The regression line (*R* ^2^ = 0.90) was calculated based on the first ten points (□).

At increasing partial pressure of CD_3_CN (Figure 1a), the ν(C≡N) region revealed the presence of hydrogen bonding adducts with BAS (2287, 2298 cm^−1^; ν(OH) region in Figure S7) and silanols (2278 cm^−1^), and Lewis acid–base complexes with LAS (2323 cm^−1^), beside physisorption (2265 cm^−1^) (Figures S7 and S8).^[^ ^18^ ^]^ Quantification indicated a loss of the original LAS by ca. 58% and an increase of BAS by ca. 14% (Figure S9, Table S2).

The carbenium ions responded very differently to the titration with CD_3_CN. While CP^+^ (1513 cm^−1^) were unperturbed, PMB^+^ (1624 cm^−1^) disappeared sharply at full saturation of the zeolite with CD_3_CN (Figure 1b) indicating their greater reactivity.

A linear relationship between the intensity of the ν(OH) of BAS and the ν(C≡N) of BAS–NCCD_3_ adducts is expected in the case the loss of BAS was only due to H‐bonding with the probe molecule. Instead, a net deviation was observed in correspondence of the points where the zeolite was close to saturation (●, second last point in red, Figure 1c) or saturated (the last point in blue, ●) with CD_3_CN and of the loss of the signal of PMB^+^ (Figure 1c). The sudden jump of the intensity of the ν(C≡N) mode of BAS–NCCD_3_ adducts right after the intensity of the ν(OH) mode of BAS was already null (last spectrum at 10 torr), needs to be interpreted by postulating the formation of additional BAS by the back‐donation of a proton from PMB^+^ to the zeolite framework forming “extra” BAS, which are then titrated by CD_3_CN at full saturation, and the corresponding neutral benzene derivative. The plausibility of such reaction sequence is supported by the computed relative stability of the intermediates (vide infra).

Reaction with methanol and formation of carbenium ions in the case of Z5 (Figure S10) caused a decrease of the BAS by ca. 16% and of the already very low fraction of LAS by ca. 40% (Figure S11, Table S2). Albeit a higher content of CP^+^ relative to PMB^+^, the reactivity of the two carbenium ions is very different mirroring the behavior observed on Z5_EFAL, and their order of reactivity was analogous despite the two different chemical environments in the two zeolites.

Deprotonation of PMB^+^ was reversible. The spectrum obtained at 298 K after evacuation of Z5_EFAL containing the carbenium ions (Figure 2b) featured the peaks at 1624 and 1490 cm^−1^ with similar intensities to those measured before exposure to CD_3_CN, as well as the unperturbed peak of CP^+^. Additionally, only the peaks of adsorbed CD_3_CN on the LAS and BAS persisted (Figure 2a).

**Figure 2:**
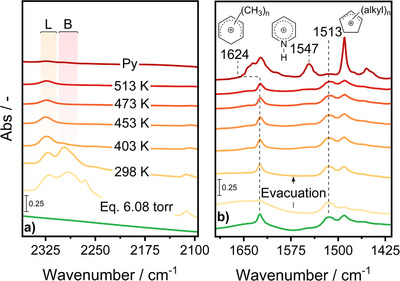
In situ FTIR spectra of methanol‐treated Z5_EFAL in the a) ν(C≡N) and b) ν(C═C) regions (bottom to top) before CD_3_CN adsorption, at full saturation with CD_3_CN (6.08 torr), after evacuation at 298, 403, 453, 473, and 513 K, and after dosing pyridine (Py; 0.05 torr). “L” and “B” stand for LAS and BAS complexed with CD_3_CN, respectively. All the spectra were collected at room temperature.

The stability of CP^+^ was probed by pyridine adsorption on Z5_EFAL after removal of adsorbed CD_3_CN at 403, 453, 473, and 513 K (Figure 2). The changes in the ν(OH) region along the same sequence are shown in Figure S12a. Both the PMB^+^ and CP^+^ remained substantially unperturbed during the thermal treatment. Pyridine (0.05 torr) caused the disappearance of the peaks of BAS and EFAL species (Figure S12a) concurrent to an intensity change of the peaks of all carbenium ions because of the interaction with the titrant. The attenuation of the peak of CP^+^ at 1513 cm^−1^ witnessed their partial disappearance likely due to the deprotonation event that did not occur with CD_3_CN, which was accompanied by the appearance of pyridinium ions from protonation on BAS (1490, 1547, and 1623 cm^−1^, Figure S12)^[^ ^19^ ^]^ and pyridine coordinated to LAS (ca. 1455 cm^−1^, Figure S12b).^[^ ^19^ ^]^ Evacuation did not restore the signals observed before pyridine adsorption demonstrating its stability in the surface‐bound state.

The relative stability of PMB^+^ and CP^+^ cations was studied by density functional theory (DFT) calculations to investigate their competitive adsorption with CD_3_CN and pyridine within the pores of a periodic structure of H‐ZSM‐5 with aluminum substitution at the T‐12 site (Section 1.3 of the Supplementary Information). We employed pentamethylbenzenium (pentaMB^+^) and hexamethylbenzenium (hexaMB^+^) ions as well as tetramethylfulvenium (tetraMF^+^) and pentamethylcyclopentenyl (pentaMCP^+^) cations as representative structures of C6 and C5 species, respectively. We chose these highly methylated model adsorbates as methylation of benzene is energetically downhill,^[^ ^20^, ^21^ ^]^ but an intermediate degree of methylation of the ring of active hydrocarbon‐pool species in H‐ZSM‐5 has also been reported.^[^ ^22^ ^]^ However, these differences in methylation degree result only in a small difference in adsorption free energy between hexaMB^+^ (9 kJ mol^−1^, Figure 3a) and pentaMB^+^ (5 kJ mol^−1^), such that our results should be more broadly representative including less substituted species.

**Figure 3:**
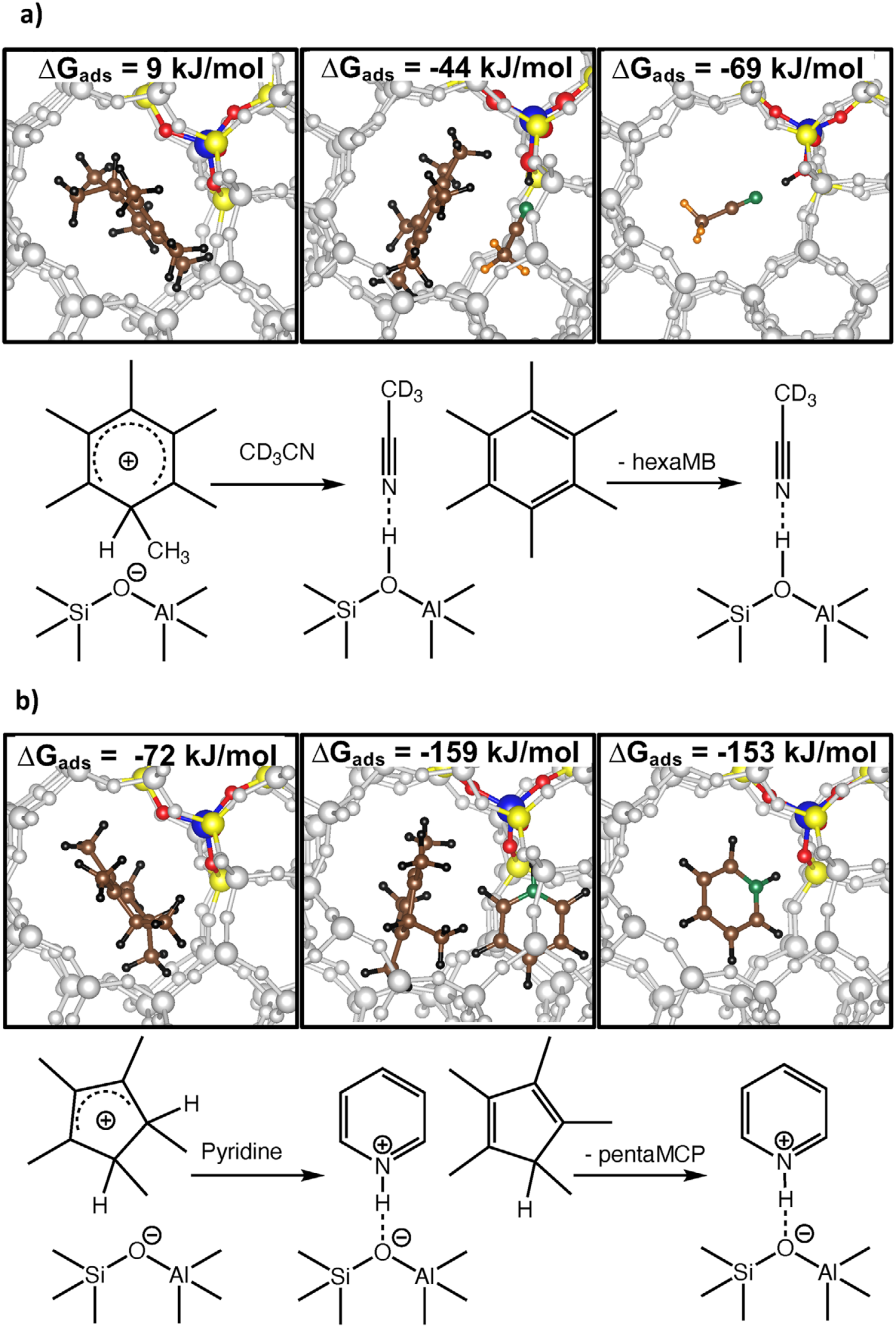
Calculated structures, free energies of adsorption (298 K, 1 bar reference pressure) and adsorption scheme of a) hexaMB with CD_3_CN and b) pentaMCP with pyridine in H‐ZSM‐5. Color code: blue, Al; red, O; yellow, Si; green, N; brown, C; black, H; orange, D; grey, framework Si and O.

Upon addition of CD_3_CN, adsorbed hexaMB^+^ is deprotonated resulting in co‐adsorption of hexaMB and CD_3_CN with a free energy change of ‐44 kJ mol^−1^ (Figure 3a). The desorption of hexaMB leads to an increase in adsorption free energy by 25 kJ mol^−1^, showing that CD_3_CN can effectively displace hexaMB^+^. These values are similar in the case of pentaMB^+^ (5, ‐35, ‐69 kJ mol^−1^; Tables S3 and S4, Figure S12). The 5‐membered ring carbenium ions adsorb much stronger (pentaMCP^+^, ‐72 kJ mol^−1^, Figure 3b; tetraMF^+^ ‐97 kJ mol^−1^) disfavoring co‐adsorption and replacement by CD_3_CN (Tables S3 and S4, Figure S12). Pyridine, however, co‐adsorbs strongly with both benzenium and 5‐membered ring ions (pentaMCP, ‐159 kJ mol^−1^; tetraMF, ‐157 kJ mol^−1^) and detachment of CP species from BAS (Figure 3b) is feasible, with similar adsorption energies before and after desorption (note that ∆*G* is calculated at 1 bar for all species). This aligns well with the experimental findings that upon pyridine adsorption the IR peaks of both types of carbenium ions disappear, whereas CD_3_CN adsorption only deprotonates the benzenium ions.

The shift of the calculated ν(C≡N) mode of CD_3_CN by + 50–60 cm^−1^ upon co‐adsorption of a hydrocarbon and CD_3_CN, and by + 40 cm^−1^ for CD_3_CN only (Table S3) is expected experimentally but is convoluted within the heterogeneous speciation of adsorption sites in the zeolite. For pyridine, no difference between single adsorption and co‐adsorption was observed. The 19b vibration (ca. 1560 –1565 cm^−1^) was in agreement with adsorption on BAS and with the experimental spectra of Figure 2. The dependence of the calculated ν(C═C) vibrations in hexaMB^+^ (1605 cm^−1^) and pentaMB^+^ (1615 cm^−1^) from the degree of methylation is also in fair agreement with the experiments. Similarly, signals calculated at 1536 (pentaMCP^+^) and 1553 cm^−1^ (tetraMF^+^) are slightly higher than the experimental value (1513 cm^−1^).

The trend of stability observed for the C5 and C6 ions is also supported by the proton affinity (PA) values of substituted benzenes and cyclopentadienes (Table S6 lists PA for various bases). Alike CD_3_CN (779.5 kJ mol^−1^), methanol (754.3 kJ mol^−1^) reacts only through H‐bonding with BAS, which maintains the hydrogen atom as long as there is no excess of the base.^[^ ^23^, ^24^ ^]^ On the other hand, ammonia (853.6 kJ mol^−1^) and pyridine (930.0 kJ mol^−1^) are protonated by BAS to form the corresponding ion coordinated to the negatively charged zeolite framework.^[^ ^25^ ^]^ The stabilization of onium ions by ionic interactions with the negatively charged framework and by van der Waals (dispersive) interaction with the zeolite walls^[^ ^26^ ^]^ is the driving force to overcome the high PA of the zeolitic BAS (1139–1204 kJ mol^−1^) and typically leads to the formation of the (meta)stable carbenium ions.^[^ ^27^ ^]^ Polymethylbenzenes exhibit PA values that approach or are higher than that of ammonia depending on the number of methyl substituents and their position: hexaMB and 1,2,4,5‐tetraMB form ions in zeolite beta and the corresponding carbenium ion exhibits a PA threshold of ca. 820 kJ mol^−1^ (Table S6).^[^ ^17^, ^28^, ^29^ ^]^ On the contrary, the calculated PA value of 1,3‐dimethylcyclopentadiene (902.1 kJ mol^−1^) is close to that of the strong base pyridine, suggesting that the stability of the corresponding cation is ca. 80 kJ mol^−1^ higher than the lower limit of PA of polymethylbenzenes, in agreement with those between C5 and C6 species found in our calculations (Figure 3, Table S3), reflecting the reactivity of the carbenium ions confined within the pores of H‐ZSM‐5. Pyridine on the other hand has a higher PA, thus being able to deprotonate C5 carbenium ions.

The different tendency of the two classes of carbenium ions toward deprotonation by a strong base or an excess of a weaker base is crucial to understand their involvement in acid catalysis and the chemistry of the MTO process. Adsorption of methanol in the presence of a polymethylbenzenium ion in the pores of the zeolite induces the deprotonation of the carbenium ion forming a polymethylbenzene, which may undergo stepwise methylation and a final dealkylation event to yield the olefin, thus restoring the carbenium ion. Methylation is possibly facilitated by the presence of an adjacent BAS.^[^ ^30^ ^]^ Since the deprotonation mechanism that yields the neutral hydrocarbon derivative is a key step in the overall reaction cycle, cyclopentenyl cations are expected to be less reactive than benzenium ions under conditions where carbenium ions are stable in the adsorbed form, and at the same partial pressure of oxygenates.

In summary, we have built polymethylbenzenium ions and alkyl‐substituted cyclopentenyl cations in situ within the pores of H‐ZSM‐5 exploiting the methanol‐to‐olefins process and have provided experimental and theoretical evidence of a scale of reactivity of carbenium ions. We have used the vibrational modes of adsorbed CD_3_CN, a probe molecule of similar proton affinity to methanol, to show that polymethylbenzenium ions deprotonate reversibly at Brønsted acid sites. The large differences in adsorption free energies of alkyl‐substituted cyclopentenyl cations confirm that they react only in the presence of a stronger base than methanol. These results outline the Brønsted acid chemistry of carbenium ions in zeolites and pave the way to their improved characterization in a reactive environment.

## Supporting Information

The authors have cited additional references within the Supporting Information.^[^ ^31^, ^32^, ^33^, ^34^, ^35^, ^36^, ^37^, ^38^, ^39^, ^40^, ^41^, ^42^, ^43^ ^]^

## Conflict of Interests

The authors declare no conflict of interest.

## Supporting information



Supporting Information

## Data Availability

The data that support the findings of this study are available from the corresponding author upon reasonable request.
