# Supplementary material for: Influence of the Acid Reactivity of Carbenium Ions in Zeolites on the Methanol‐to‐Olefins Process
Source: Angew Chem Int Ed Engl. 2025 Oct 6;64(47):e202514759. doi: 10.1002/anie.202514759 (PMC12624309; doi:10.1002/anie.202514759)
Supplement: Supplementary file 1 — Supporting Information [file ANIE-64-e202514759-s001.docx]

Supplementary Information

Influence of the acid reactivity of carbenium ions in zeolites on the

methanol-to-olefins process

Luca Maggiulli,^a,b^ Vitaly L. Sushkevich,^a^ , Annika E. Enss,^c^ Felix Studt,^c,d^ Jeroen A. van Bokhoven,*^a,b^ Davide Ferri*^a^

^a^ Paul Scherrer Institute, PSI Center for Energy and Environmental Sciences, CH-5232, Villigen PSI, Switzerland

^b^ Institute for Chemical and Bioengineering, ETH Zurich, Vladimir-Prelog-Weg 1, CH-8093 Zurich, Switzerland

^c^ Institute for Catalysis Research and Technology, Karlsruhe Institute for Technology, Hermann-von-Helmholtz-Platz 1, 76344 Eggenstein-Leopoldshafen, Germany

^d^ Institute for Technical and Polymer Chemistry, Karlsruhe Institute for Technology, Engesserstr. 18 /20, 76131 Karlsruhe, Germany

* Corresponding authors

Jeroen A. van Bokhoven, e-mail: jeroen.vanbokhoven@chem.ethz.ch

Davide Ferri, e-mail: davide.ferri@psi.ch

**1. Experimental section**

**1.1. Materials preparation and characterization**

Zeolite ZSM-5 (MFI, Si/Al = 11.5; Zeolyst) was purchased in the ammonium form and was subjected to two different thermal treatments: 1) Z5_EFAl was obtained after thermal treatment in static air at 823 K for 6 h (2 K/min) in a muffle oven; 2) in situ activation inside the spectroscopic cell under inert atmosphere (Ar or vacuum) at 673 K for 8 h (1 K/min) followed by temperature adjustment to reaction temperature for testing without prior exposure to air delivered sample Z5. Z5_EFAl was activated before testing at 673 K for 1 h (10 K/min) inside the spectroscopic cell and then brought to reaction temperature. Characterization of the Z5_EFAl sample is provided in Figure S1-3, Table S1 and our previous work.^1^

Methanol (>99,9 %; OPTIMA LC/MS grade, Thermo Fischer Scientific) was used as received.

**1.2. In situ transmission FTIR of CD_3_CN adsorption**

Transmission FTIR measurements were carried out using a iS50 spectrometer (Thermo Scientific) equipped with a deuterated triglycine sulfate (DTGS) detector and a home-built quartz cell, which enables high-temperature treatment. The analysis was carried out to quantify the changes in BAS/Lewis acid sites (LAS) ratio after an in situ treatment in methanol that led to the formation of carbenium ions. A weighted amount of sample was pressed into a self-supporting wafer (2 cm^2^), placed into the quartz cell, and subjected to the activation protocol. Z5_EFAl underwent a thermal treatment under vacuum at 823 K (10 K/min) for 5 h before reaction with methanol; Z5 was generated in situ under vacuum starting from ZSM-5 in ammonium form at 673 K (1 K/min) for 8 h. After lowering the T to 543 K, methanol (10 torr) was dosed for 13 min on Z5_EFAL and 23 min on Z5 to generate the carbenium ions. FTIR spectra of adsorbed CD_3_CN were obtained on Z5_EFAL and Z5 on the pristine material and after methanol treatment at room temperature. Calibrated aliquots (1.6 ml) of gaseous CD_3_CN (at 1, 2, 4 and 10 torr) were dosed followed by complete saturation with final pressure of 5.8 torr (Z5_EFAl pristine), 6.08 torr (Z5_EFAl methanol treated), 6.0 torr (Z5 pristine), 6.67 torr (Z5 methanol treated). The spectra were collected at room temperature by accumulating 32 scans at a spectral resolution of 4 cm^-1^. The difference spectra were obtained by subtracting the spectrum after activation from the spectra of the zeolite with adsorbates. The peak fitting of the final spectra was carried out using the Omnic 9.5.9 software package, employing Voigt shape for the peaks. The quantification of Brønsted acid sites was carried out using the Beer-Lambert law assuming an integrated extinction coefficient of the ν(CN) mode of the (BAS)OH∙∙∙NCCD_3_ adduct at ca. 2287-2299 cm^-1^ of 2.05 cm/µmol.^2^ Lewis acid sites quantification was performed using the extinction coefficient of the ν(CN) mode of the (LAS)∙∙∙NCCD_3_ adduct at 2325 cm^-1^ (3.60 cm/µmol).^2^ Absorption peaks at ca. 2250 cm^-1^ and ca. 2214 cm^-1^ for the pristine Z5 and Z5_EFAL (Figures S4 and S5) and exposed Z5 and Z5_EFAL (Figures S11 and S9) are associated to the asymmetric and symmetric stretching modes of the deuterated methyl group of CD_3_CN and are not further discussed in the main text.

After saturating the sample Z5_EFAl with CD_3_CN, the cell was evacuated to observe the change of the hydrocarbon surface species as a consequence of the removal of physisorbed CD_3_CN. The sample was then heated to increasing temperatures of 403, 453, 473, and 513 K to remove the chemisorbed CD_3_CN from Brønsted and Lewis acid sites. Finally, pyridine (0.05 torr) was dosed. All the spectra were collected at room temperature after the heating treatments and along the adsorption.

**1.3. Computational methods**

The structure of the H-ZSM-5 unit cell with a Si/Al ratio of 35 was taken from previous work,^3^ the T12 site was used as Brønsted acid site. Structure optimizations were carried out using the Vienna Ab Initio Simulation Package (VASP) in the atomic simulation framework (ASE)^4^ with the dispersion-corrected PBE-D3 functional^5, 6^ and the projector-augmented wave method.^7^ The energy cutoff was set to 400 eV, convergence criterion to 0.001 eV/Å, and *k*-point sampling was only performed at the Γ-point. As representative polymethylbenzenes (MB), hexaMB and pentaMB were chosen. Pentamethylcyclopentadiene (pentaMCP) and tetramethylfulvene (tetraMF) were chosen as 5-ring species. The adsorbed structures of hexaMB^+^, pentaMB^+^ and tetraMF^+^ were taken from previous work.^8^ For vibrational analysis, a partial Hessian including the acid site and adsorbate was calculated. Adsorption free energies at the temperature of the FTIR spectra (298 K) were calculated with the harmonic-oscillator, rigid-rotator and free-translator approximations. Frequencies below 12 cm^-1^ were raised to that value to avoid larger inaccuracies in the harmonic-oscillator approximation.^9, 10^ To calculate the vibrations of CD_3_CN, structures were first optimized for CH_3_CN and hydrogen masses set to 2.0 for CD_3_CN vibrations. The vibrations of acetonitrile and pyridine were multiplied by a scaling factor, to account for the differences of computational and experimental values. The scaling factor is calculated as $Scaling factor= \frac{\nu^{\mathrm{experimental}}}{\nu^{\mathrm{calculated}}}$.

For the acetonitrile C-N vibration the scaling factor is 2278 cm^-1^ / 2297 cm^-1^ = 0.992, and for the pyridine 19b vibration 1440 cm^-1^ / 1427 cm^-1^ = 1.009.

**
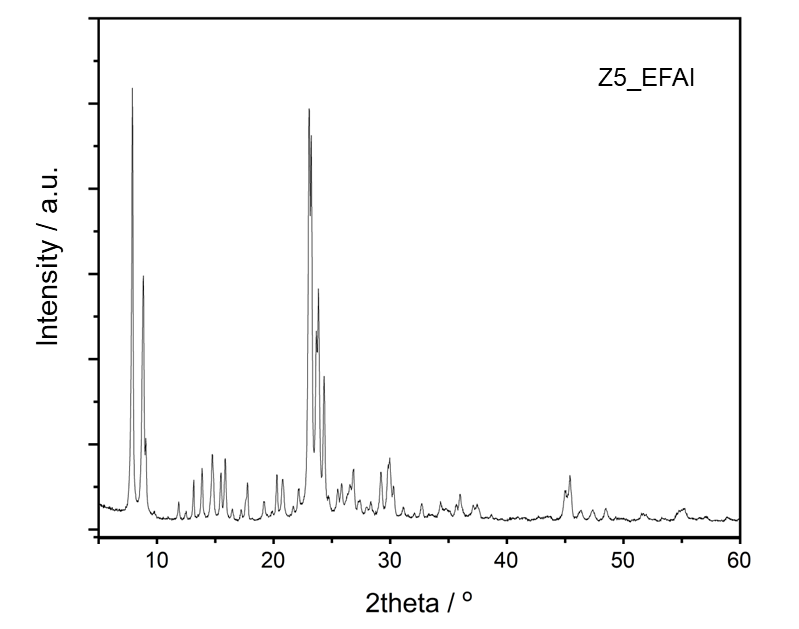
**

**Figure S1**. Powder XRD pattern of Z5_EFAl. Typical reflections of respective topologies were observed by comparison with simulated diffraction patterns.^11^


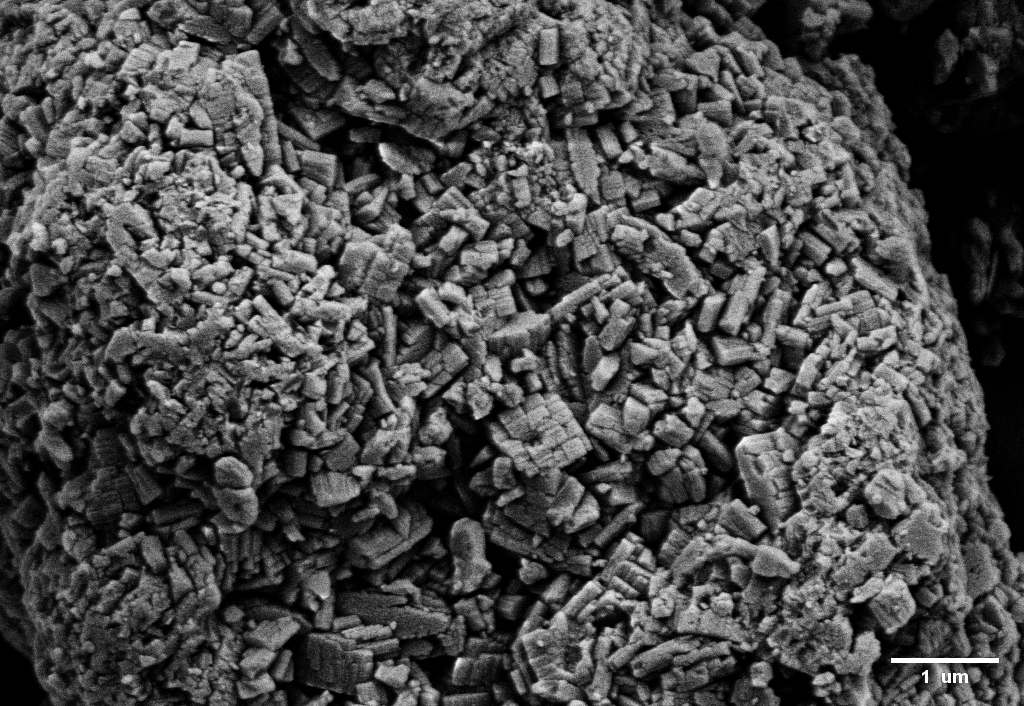


**Figure S2**: SEM micrograph of Z5_EFAl.

**Table S1**: Textural properties of Z5_EFAl measured by Ar physisorption at 87 K.^[a]^

|  | BET value (m^2^/g) | Total pore volume (cc/g) | Micropore volume (cc/g) | Micropore area (m^2^/g) | External area (m^2^/g) |
| --- | --- | --- | --- | --- | --- |
| ZSM-5 | 360.2 | 0.19 | 0.113 | 320.6 | 39.6 |

^[a]^ The BET range was chosen following the micropore assistant based on Rouquerol criteria^12^ in the ASiQ software. The total pore volume was calculated using the last point of adsorption isotherm. The micropore volume and area as well as external area were extracted by using the t-plot method and generalized Halsey equation.


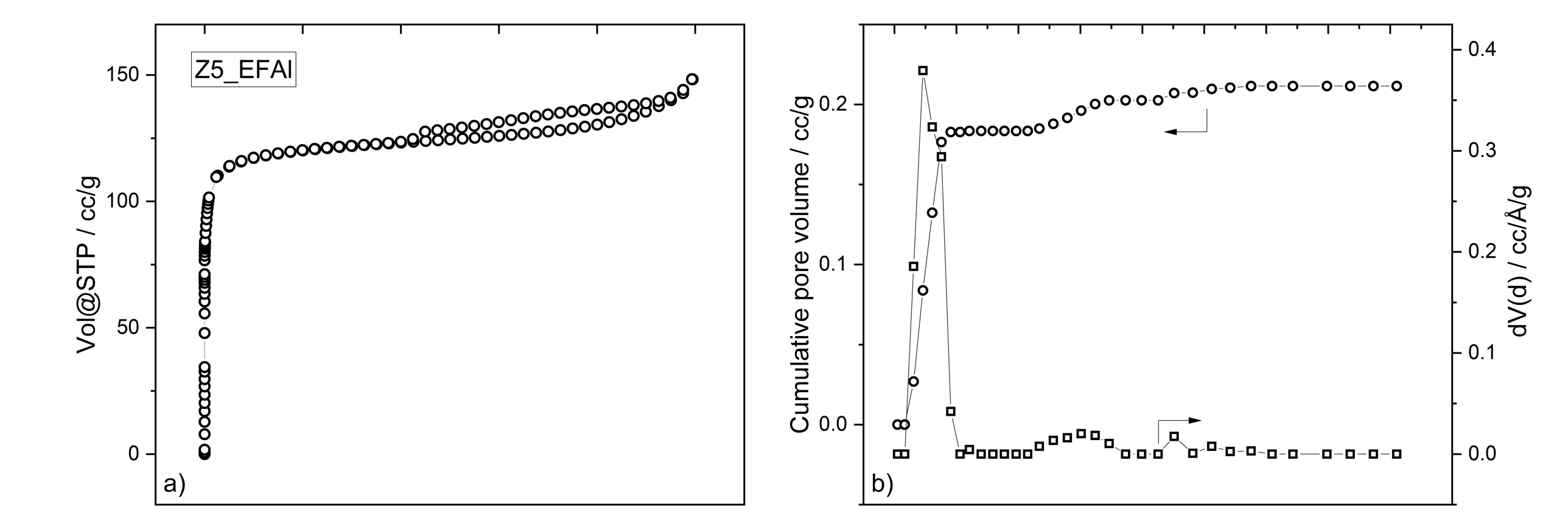


**Figure S3**: Ar physisorption isotherm of Z5_EFAl (a) and micropore size distribution calculated using NLDFT with cylindrical pore model (b). Type I isotherms with a steep Ar uptake and H4-type hysteresis. The initial uptake at low relative pressure of the adsorption isotherms highlights the microporosity of the materials. The cumulative pore volume and its first derivative point out the prevalence of small micropores, which are on average of 4.9 Å.


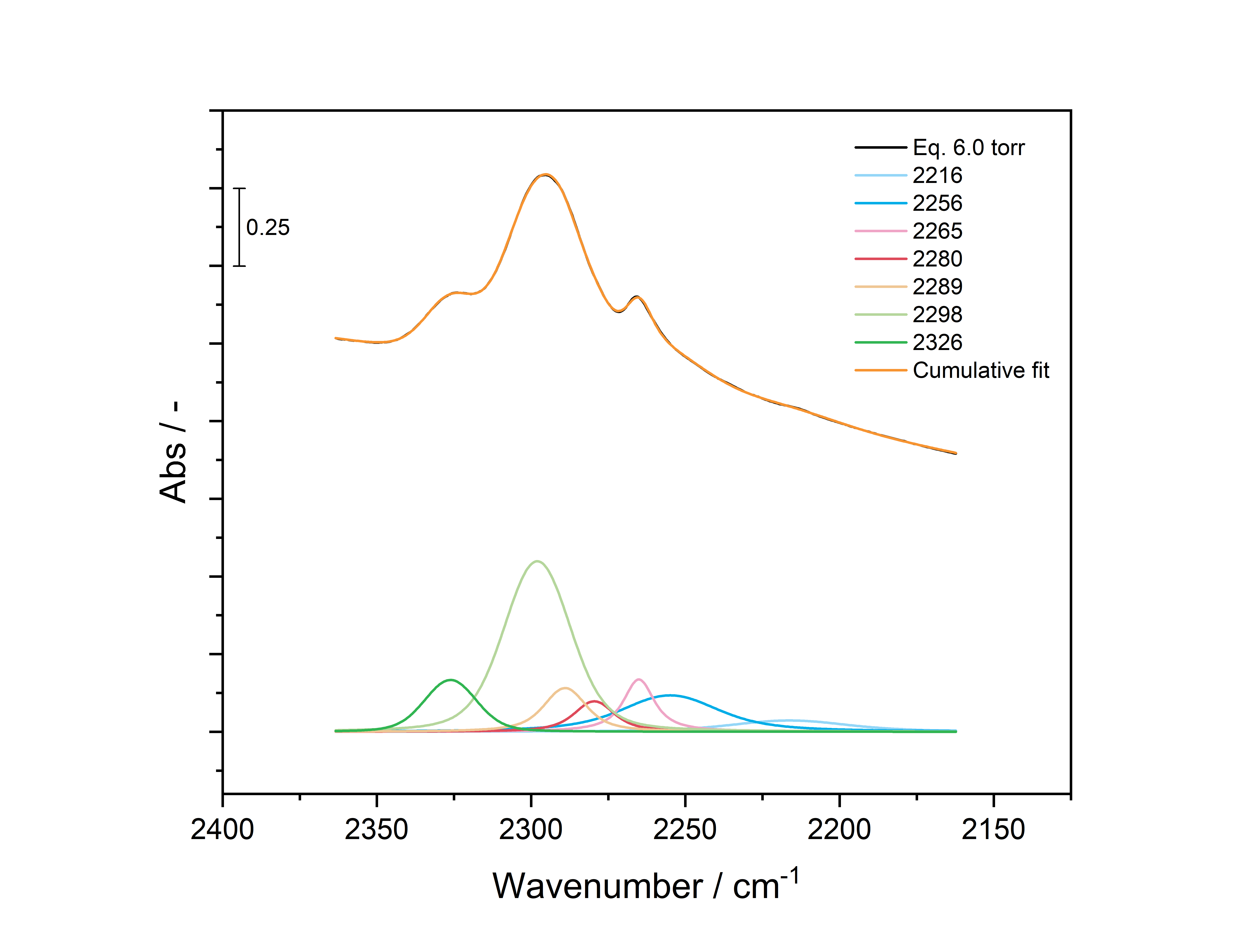


**Figure S4**. Peak fitting of the ν(C≡N) region of the FTIR spectrum collected on Z5 after saturation with CD_3_CN. The areas of the peaks were used to calculate the acid sites concentration reported in Table S2.


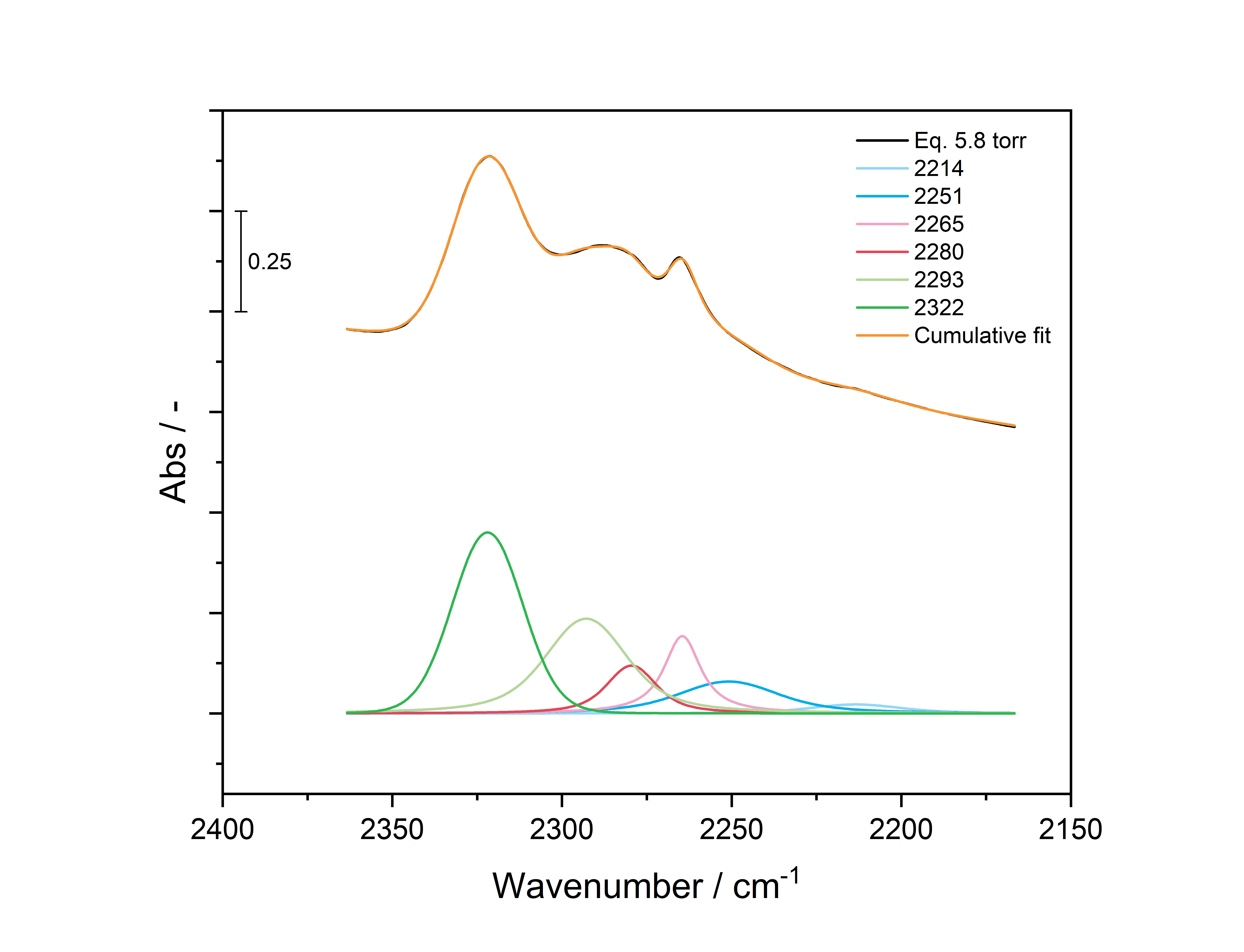


**Figure S5**. Peak fitting of the ν(C≡N) region of the FTIR spectrum collected on Z5_EFAl after saturation with CD_3_CN. The areas of the peaks were used to calculate the acid sites concentration reported in Table S2.

**
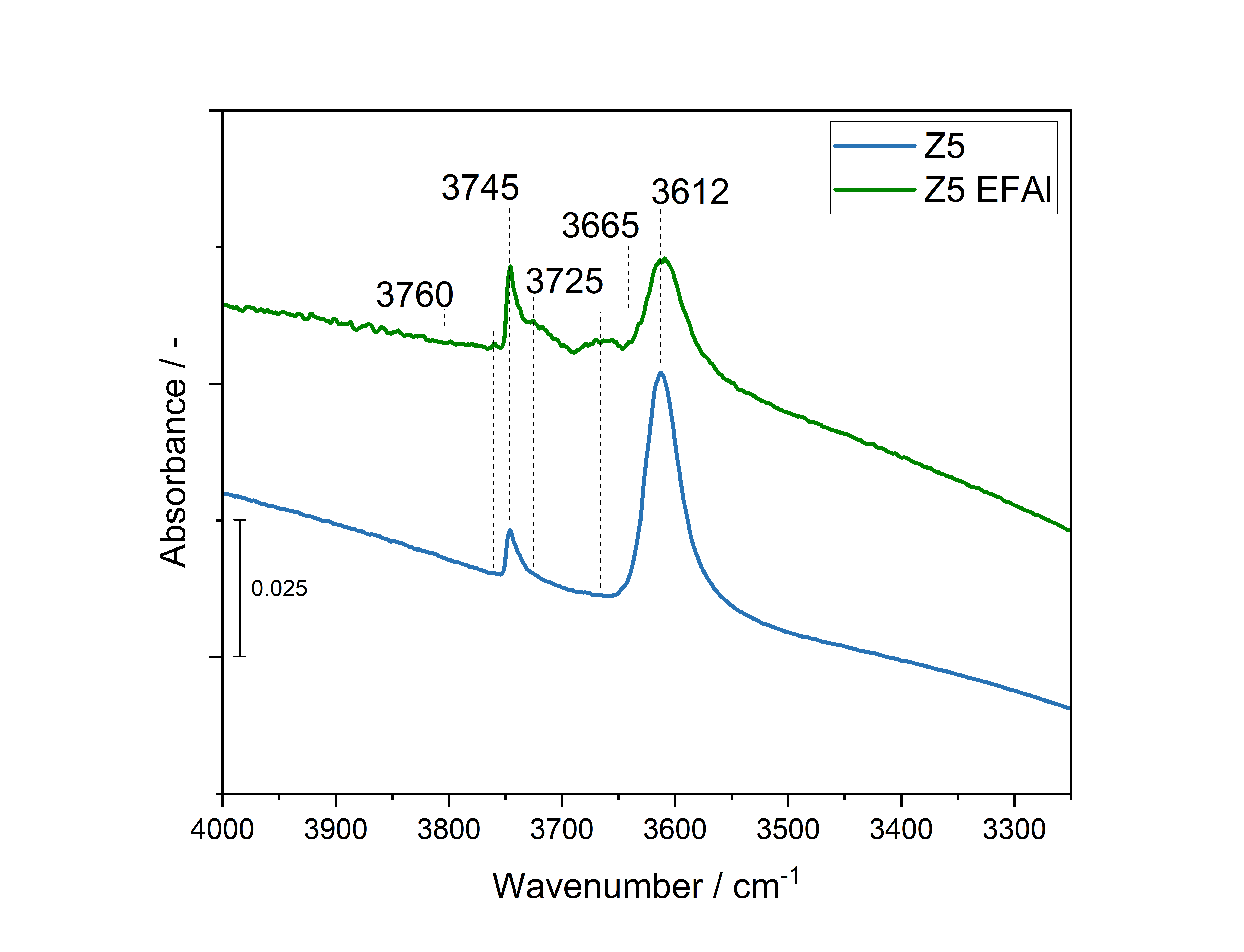
**

**Figure S6**. Comparison of the ν(O-H) region of the transmission FTIR spectra of Z5 and Z5_EFAl. A discussion of the peak assignment is given in the main text.

**Table S2**. Concentration of BAS and LAS obtained by CD_3_CN titration of pristine and methanol-treated Z5 and Z5_EFAl. Peak fitting was performed considering that the areas of the BAS – NCCD_3_ and Si-OH – NCCD_3_ adducts follow the ratio of the ν(OH) peak of BAS (1:2) and Si-OH (1.35:1) of Z5_EFAl relative to Z5.

| Sample | BAS^a^ (µmol/g) | ν(C≡N) BAS⸱⸱⸱NCCD_3_ (cm^-1^) | LAS^b^ (µmol/g) | ν(C≡N) LAS⸱⸱⸱NCCD_3_ (cm^-1^) |
| --- | --- | --- | --- | --- |
| Z5_EFAl | 766 | 2294 | 590 | 2322 |
| Z5_EFAl MeOH react. | 873 | 2298, 2287 | 249 | 2321 |
| Z5 | 1645 | 2298, 2289 | 181 | 2326 |
| Z5 MeOH react. | 1390 | 2299, 2290 | 108 | 2323 |

^a^ Brønsted acid sites

^b^ Lewis acid sites


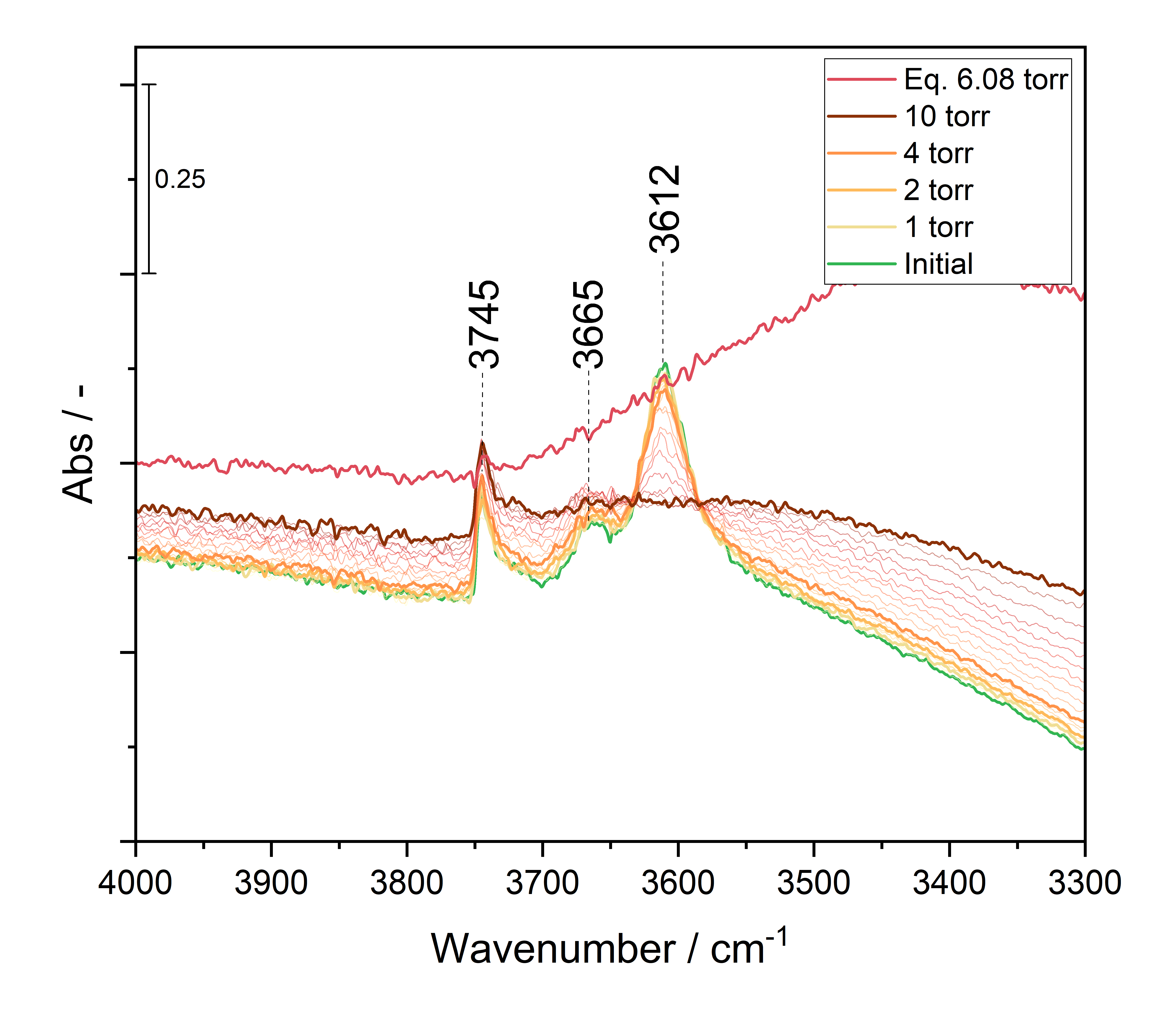


**Figure S7**. IR peaks in the ν(O-H) region during the CD_3_CN titration of Z5_EFAL after reaction with methanol at 543 K. A discussion of the peak assignment is given in the main text.


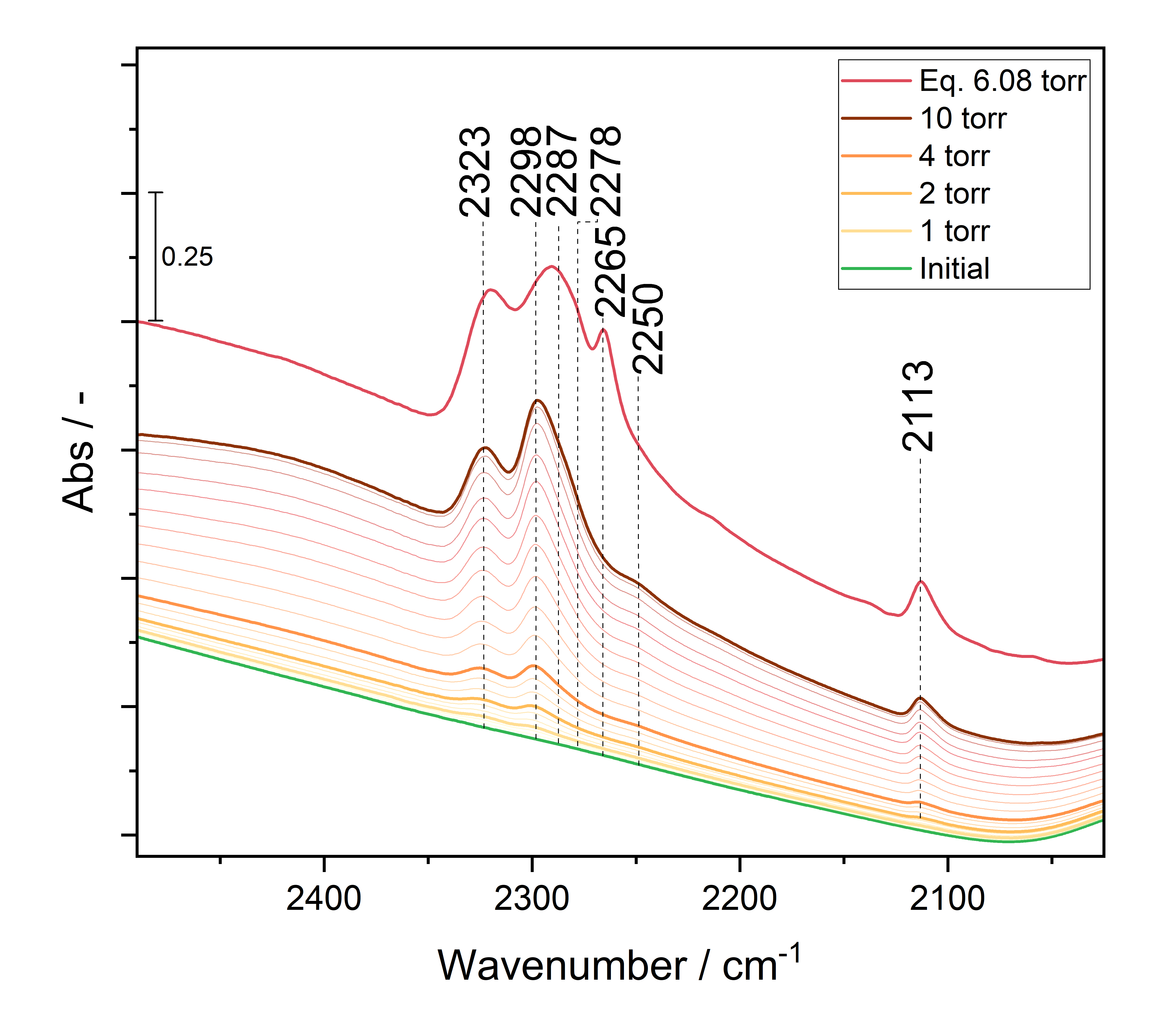


**Figure S8**. IR peaks in the ν(C≡N) region during the CD_3_CN titration of Z5_EFAL after reaction with methanol at 543 K. A discussion of the peak assignment is given in the main text.


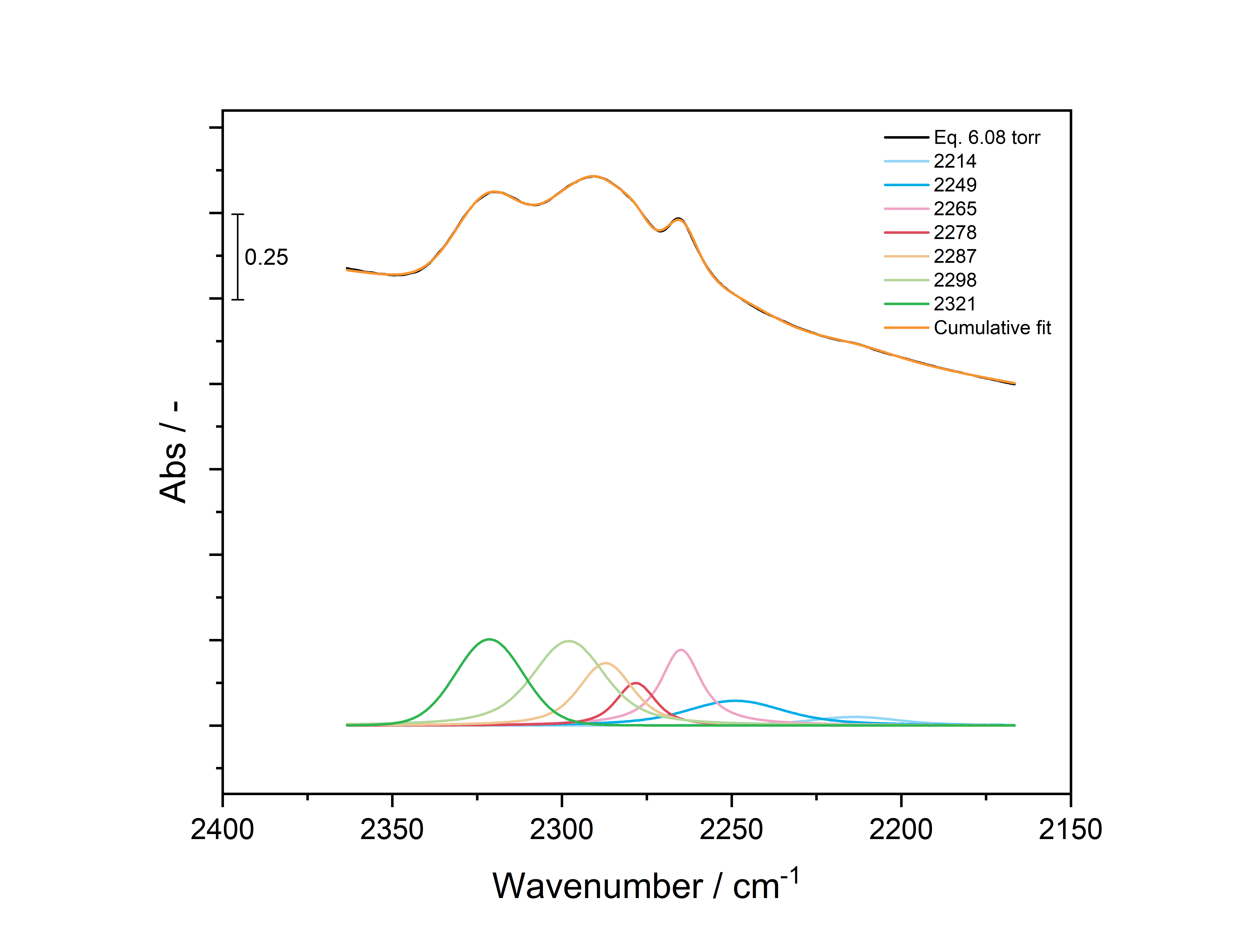


**Figure S9**. Peak fitting of the ν(C≡N) region of the transmission FTIR spectrum collected on methanol-treated Z5_EFAl after saturation with CD_3_CN. The areas of the peaks were used to calculate the acid sites concentration reported in Table S2.


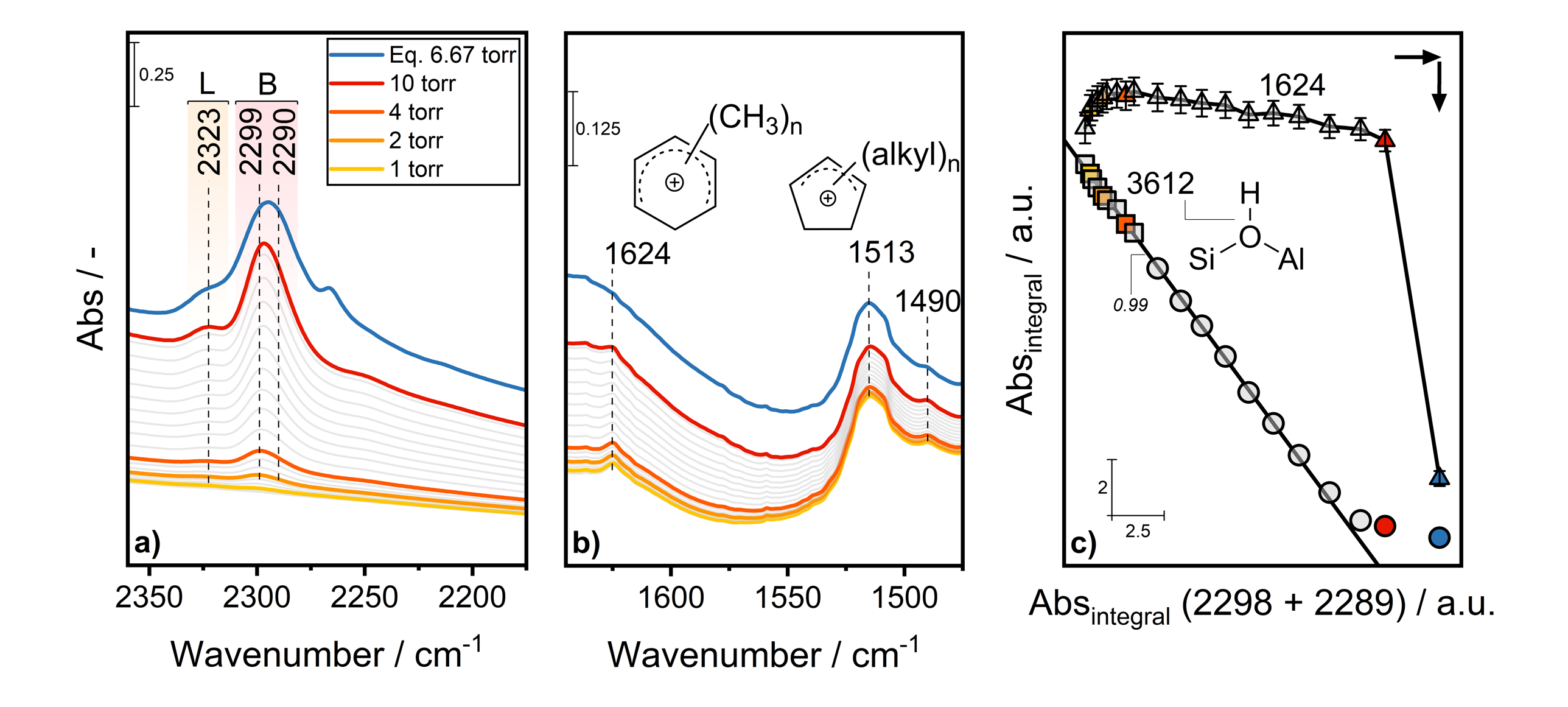


**Figure S10.** In situ FTIR spectra of methanol-treated Z5 in the (a) ν(C≡N) and (b) ν(C=C) regions during the CD_3_CN titration of the acid sites. Labels “L” and “B” stand for LAS and BAS complexed with CD_3_CN, respectively. (c) Correlation between the areas of the bands of the ν(O-H) mode of the BAS (3612 cm^-1^) and of the ν(C-C=C)^+^ mode of polymethylbenzenium ions (1624 cm^-1^) with the areas of the bands of the ν(C≡N) mode of the BAS – NCCD_3_ complex (Abs_integral_(2298+2289)). The regression line was calculated based on the first ten points (□) and the R^2^ coefficient is indicated.


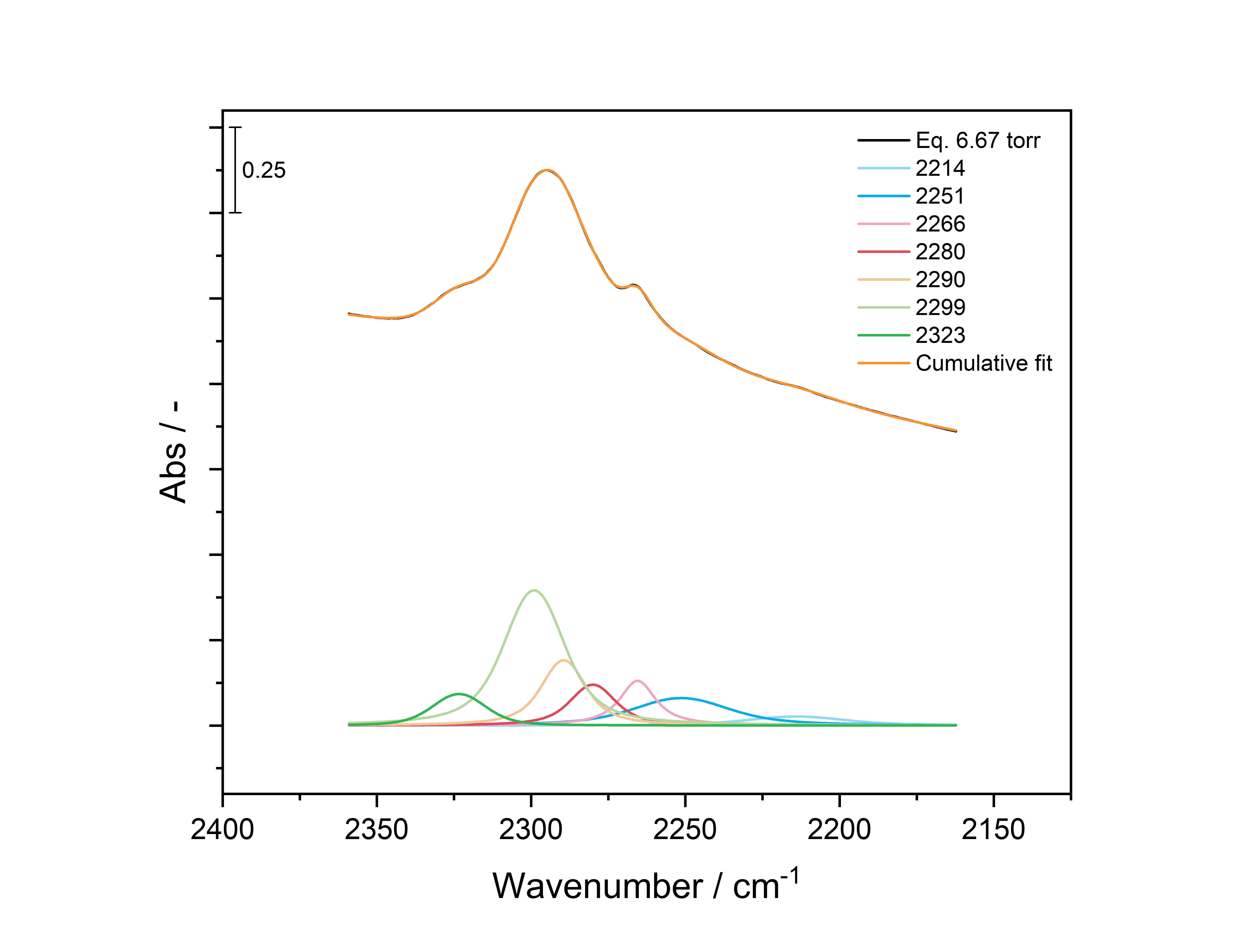


**Figure S11**. Peak fitting of the ν(C≡N) region of the transmission FTIR spectrum collected on methanol-treated Z5 after saturation with CD_3_CN. The areas of the peaks were used to calculate the acid sites concentration reported in Table S2.


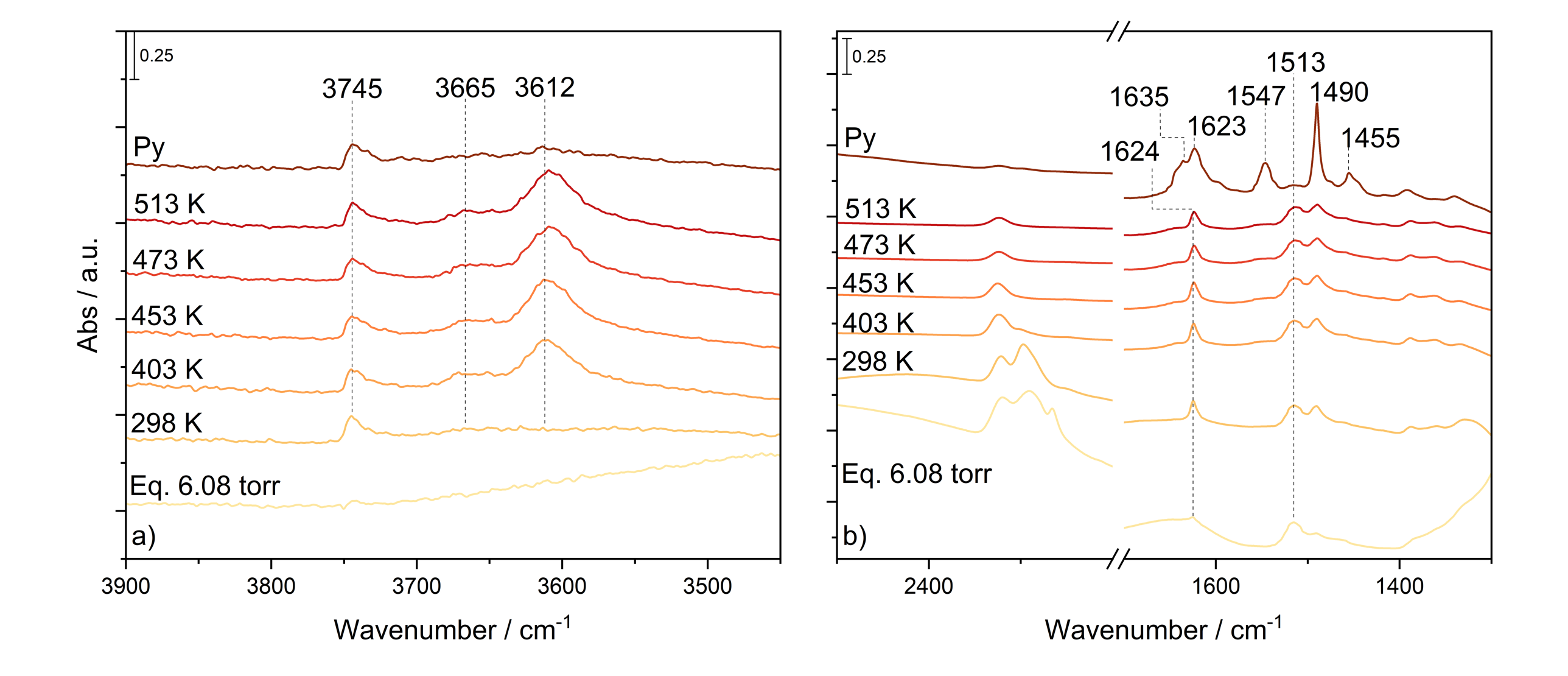


**Figure S12**. IR spectra peaks in the (a) ν(O-H), and (b) ν(C≡N) and ν(C=C) regions after heat treatment at increasing temperatures and after the final dosage of pyridine (0.05 torr) over Z5_EFAL.

**Table S3**. Adsorption free energies at 298 K for all investigated adsorbed and co-adsorbed species. Where applicable, vibrational frequencies of CD_3_CN C-N stretch, pyridine 19b vibration and carbenium ion ring stretches are provided. The frequencies of CD_3_CN and pyridine were scaled to better match experimental results (see Computational Method section above).

| Adsorbate | ∆G_ads_ (298K) in H-ZSM-5  [kJ/mol] | ∆E_ads_ (0K) in H-ZSM-5  [kJ/mol] | ν [cm^-1^]  C-N stretch  [scaled] | ν [cm^-1^]  Pyridine 19b  [scaled] | ν [cm^-1^]  Ring stretches  [unscaled] |
| --- | --- | --- | --- | --- | --- |
| CD_3_CN | -69 | -113 | 2320 |  |  |
| Pyridine | -153 | -205 |  | 1563 |  |
| hexaMB^+^ | 9 | -65 |  |  | 1604 |
| pentaMB^+^ | 5 | -58 |  |  | 1615 |
| tetraMF^+^ | -97 | -158 |  |  | 1553 |
| pentaMCP^+^ | -72 | -137 |  |  | 1536 |
| CD_3_CN + hexaMB | -44 | -160 | 2339 |  |  |
| CD_3_CN + pentaMB | -35 | -148 | 2339 |  |  |
| CD_3_CN + tetraMF | -67 | -175 | 2341 |  |  |
| CD_3_CN + pentaMCP | -83 | -190 | 2332 |  |  |
| Pyridine + hexaMB | -120 | -248 |  | 1565 |  |
| Pyridine + pentaMB | -110 | -233 |  | 1565 |  |
| Pyridine + tetraMF | -157 | -273 |  | 1566 |  |
| Pyridine + pentaMCP | -159 | -277 |  | 1559 |  |

**Figure S13**. Structures of adsorbed carbenium ions and co-adsorbed hydrocarbons with CD_3_CN or pyridine not shown in the main text. a) pentaMB^+^ b) tetraMF^+^. CD_3_CN co-adsorption with c) pentaMB, d) tetraMF, e) pentaMCP. Pyridine co-adsorption with f) hexaMB, g) pentaMB, h) tetraMF. Color code: blue: Al, red: O, yellow: Si, green: N, brown: C, black: H, orange: D, grey: framework Si & O.

**Table S4**. Absolute energies, ZPVE and free energies at 298 K for all investigated species.

| System | PBE-D3 | ZPVE | G(298K) |
| --- | --- | --- | --- |
| Gas phase |  |  |  |
| hexaMB_gas | -175.749 | 7.018 | -169.847 |
| tetraMF_gas | -141.533 | 5.504 | -137.022 |
| pentaMB_gas | -159.303 | 6.299 | -154.034 |
| pentaMCP_gas | -149.637 | 6.114 | -144.544 |
| CD_3_CN_gas | -36.722 | 0.953 | -36.406 |
| pyridine_gas | -71.32 | 2.348 | -69.708 |
| H-ZSM-5 single adsorbate |  |  |  |
| mfi_empty | -2301.732 | 1.248 | -2300.6 |
| CD_3_CN | -2339.641 | 2.217 | -2337.722 |
| pyridine | -2375.263 | 3.678 | -2371.896 |
| hexaMB+ | -2478.312 | 8.421 | -2470.352 |
| pentaMB+ | -2461.714 | 7.627 | -2454.579 |
| tetraMF+ | -2445.008 | 6.853 | -2438.627 |
| pentaMCP+ | -2452.906 | 7.477 | -2445.896 |
| H-ZSM-5 co-adsorbates |  |  |  |
| hexaMB_CD_3_CN | -2516.026 | 9.387 | -2507.305 |
| pentaMB_CD_3_CN | -2499.429 | 8.641 | -2491.405 |
| tetraMF_CD_3_CN | -2481.908 | 7.807 | -2474.72 |
| pentaMCP_CD_3_CN | -2490.148 | 8.404 | -2482.411 |
| hexaMB_pyridine | -2551.627 | 10.87 | -2541.401 |
| pentaMB_pyridine | -2535.005 | 10.128 | -2525.48 |
| tetraMF_pyridine | -2517.604 | 9.29 | -2508.952 |
| pentaMCP_pyridine | -2525.761 | 9.907 | -2516.495 |

**Table S5**. Vibrational frequencies for all investigated species.

| System | Frequencies |
| --- | --- |
| Gas phase |  |
| hexaMB_gas | 42.0, 57.7, 61.1, 68.6, 77.2, 81.9, 115.6, 123.1, 161.2, 189.9, 335.9, 338.6, 340.4, 342.2, 397.8, 399.4, 438.1, 444.7, 445.9, 548.2, 567.5, 568.7, 573.0, 583.6, 719.8, 794.3, 796.1, 955.1, 955.6, 964.6, 965.6, 989.2, 989.5, 1018.3, 1018.5, 1025.3, 1044.3, 1044.5, 1064.0, 1064.3, 1071.0, 1235.4, 1303.8, 1316.0, 1347.8, 1348.0, 1352.1, 1354.6, 1355.1, 1362.8, 1398.0, 1398.2, 1413.5, 1413.6, 1420.9, 1427.6, 1439.0, 1439.3, 1439.9, 1455.4, 1455.8, 1465.8, 1466.1, 1479.8, 1571.2, 1572.2, 2943.2, 2943.8, 2943.9, 2944.7, 2944.9, 2946.4, 3018.2, 3019.8, 3020.2, 3024.8, 3024.9, 3028.5, 3046.3, 3051.2, 3051.5, 3057.6, 3057.9, 3060.0 |
| tetraMF_gas | 90.2, 102.3, 117.0, 124.9, 134.1, 159.3, 180.5, 256.8, 276.9, 281.2, 294.1, 295.6, 384.3, 506.3, 542.1, 553.0, 583.4, 602.4, 684.0, 719.0, 785.9, 809.9, 897.9, 919.2, 930.2, 990.7, 998.1, 1019.5, 1021.2, 1056.0, 1073.6, 1080.7, 1132.6, 1200.7, 1307.3, 1317.5, 1356.3, 1357.9, 1365.4, 1369.8, 1395.0, 1415.2, 1420.8, 1421.9, 1422.7, 1427.3, 1435.5, 1436.8, 1440.5, 1601.4, 1638.2, 1645.6, 2942.0, 2942.8, 2943.2, 2947.1, 2985.4, 2986.1, 2986.6, 2990.3, 3038.4, 3039.0, 3043.5, 3045.7, 3063.7, 3147.4 |
| pentaMB_gas | 42.4, 98.1, 116.2, 128.0, 138.8, 167.1, 170.5, 192.6, 285.2, 294.9, 324.1, 341.4, 343.6, 378.0, 437.1, 480.2, 519.7, 544.7, 566.5, 570.1, 677.9, 713.0, 796.6, 842.8, 887.6, 965.4, 974.8, 976.2, 992.9, 998.4, 1011.7, 1021.0, 1024.5, 1056.1, 1062.9, 1065.4, 1208.9, 1226.1, 1291.3, 1321.2, 1349.8, 1350.5, 1353.0, 1356.8, 1362.1, 1390.8, 1410.0, 1416.1, 1421.7, 1424.0, 1433.5, 1444.2, 1444.8, 1447.4, 1455.3, 1462.8, 1474.1, 1568.0, 1597.6, 2947.9, 2949.3, 2950.6, 2951.4, 2954.5, 2998.0, 3002.5, 3003.4, 3005.4, 3007.5, 3038.5, 3039.5, 3064.2, 3064.7, 3067.4, 3069.2 |
| pentaMCP_gas | 99.1, 101.3, 112.9, 125.2, 142.8, 157.5, 176.9, 208.9, 258.7, 264.5, 281.2, 289.4, 353.7, 356.1, 491.4, 537.9, 547.9, 568.5, 627.6, 680.1, 777.5, 813.1, 923.7, 933.5, 950.2, 976.9, 996.3, 1016.2, 1020.8, 1041.6, 1051.1, 1063.9, 1112.1, 1124.5, 1145.2, 1192.7, 1256.4, 1297.7, 1317.9, 1344.1, 1356.6, 1358.9, 1359.3, 1367.0, 1418.4, 1419.5, 1421.3, 1424.0, 1429.8, 1431.0, 1432.2, 1440.6, 1442.2, 1448.4, 1606.6, 1659.2, 2905.5, 2933.3, 2934.9, 2944.9, 2947.7, 2958.3, 2973.2, 2974.2, 2989.8, 2992.6, 3030.9, 3033.5, 3034.5, 3035.4, 3042.5, 3044.2 |
| CD_3_CN_gas | 335.8, 336.9, 826.1, 826.8, 827.7, 1019.3, 1020.4, 1091.3, 2145.3, 2265.3, 2266.6, 2297.0 |
| pyridine_gas | 357.5, 399.1, 593.1, 646.6, 692.4, 740.4, 868.2, 922.2, 965.4, 981.1, 982.8, 1024.2, 1051.6, 1067.2, 1139.6, 1209.4, 1299.0, 1336.2, 1427.0, 1463.1, 1575.5, 1578.1, 3066.3, 3068.7, 3094.4, 3109.4, 3118.2 |
| H-ZSM-5 single adsorbate |  |
| mfi_empty | 165.7, 204.5, 206.6, 215.1, 259.3, 273.8, 287.4, 299.5, 335.0, 344.7, 373.6, 389.7, 520.7, 588.7, 641.9, 644.0, 655.6, 656.5, 664.1, 670.2, 692.0, 702.6, 723.8, 737.9, 785.3, 1070.0, 1090.6, 1094.2, 1136.2, 3699.7 |
| CD_3_CN | 42.2, 63.1, 66.3, 78.0, 101.7, 140.4, 200.4, 207.9, 214.3, 242.9, 269.6, 274.6, 299.4, 327.4, 351.4, 354.0, 356.5, 362.3, 401.6, 554.5, 614.8, 630.2, 643.5, 652.7, 656.1, 661.6, 671.1, 684.5, 700.1, 707.6, 727.7, 828.8, 831.3, 839.1, 865.4, 956.5, 1010.5, 1012.7, 1086.1, 1096.7, 1107.2, 1146.9, 1352.2, 2140.0, 2262.9, 2268.4, 2339.8, 2363.3 |
| pyridine | 39.3, 47.7, 66.5, 81.3, 102.1, 103.4, 203.7, 212.7, 220.2, 241.4, 277.4, 281.0, 299.8, 341.7, 353.5, 365.3, 393.2, 398.4, 417.5, 547.9, 597.7, 632.1, 632.6, 636.1, 636.8, 643.5, 648.7, 654.7, 660.5, 666.5, 678.3, 683.9, 685.8, 709.0, 748.2, 868.1, 935.0, 982.0, 988.1, 1001.2, 1008.9, 1020.2, 1059.5, 1066.5, 1083.1, 1108.6, 1116.9, 1150.5, 1160.8, 1196.3, 1301.6, 1345.1, 1400.9, 1475.6, 1549.3, 1623.8, 1639.4, 2687.3, 3109.8, 3117.7, 3136.1, 3138.9, 3150.8 |
| hexaMB+ | 64.7, 68.1, 85.4, 89.1, 101.2, 103.0, 121.6, 125.2, 146.3, 172.6, 191.8, 200.3, 207.3, 208.9, 216.7, 220.2, 229.5, 240.6, 259.0, 284.0, 286.0, 289.4, 299.7, 313.3, 325.5, 343.6, 345.4, 350.4, 353.0, 370.4, 375.7, 390.9, 391.6, 422.8, 439.4, 462.1, 545.4, 546.2, 554.3, 571.7, 592.0, 622.8, 626.4, 629.3, 630.9, 631.7, 643.0, 648.7, 650.6, 656.7, 680.5, 687.3, 690.3, 702.9, 801.8, 838.6, 912.9, 949.9, 955.4, 956.8, 967.1, 979.4, 987.4, 1004.5, 1020.1, 1032.3, 1047.3, 1048.2, 1068.0, 1075.5, 1088.1, 1095.7, 1101.8, 1148.2, 1161.6, 1166.6, 1185.4, 1263.2, 1327.7, 1331.8, 1343.1, 1343.8, 1361.7, 1364.9, 1371.3, 1374.3, 1403.2, 1404.5, 1409.9, 1414.2, 1418.3, 1431.4, 1439.4, 1444.6, 1445.7, 1452.6, 1455.6, 1465.7, 1492.9, 1528.1, 1604.2, 2947.7, 2951.4, 2963.6, 2964.1, 2983.7, 2988.3, 3005.3, 3007.3, 3017.3, 3032.7, 3036.5, 3043.0, 3073.0, 3104.2, 3128.6, 3137.2, 3162.5, 3189.8, 3208.8 |
| pentaMB+ | 48.4, 62.2, 64.5, 70.5, 78.6, 88.6, 105.5, 107.7, 113.3, 148.9, 170.0, 186.3, 203.6, 205.7, 210.0, 213.8, 218.0, 235.3, 258.9, 272.9, 276.8, 292.3, 310.0, 327.5, 334.6, 349.5, 350.5, 361.4, 372.4, 382.5, 398.4, 434.2, 479.5, 483.3, 531.7, 553.3, 569.9, 594.1, 622.7, 627.2, 627.9, 635.2, 638.3, 643.0, 656.2, 657.6, 672.4, 684.1, 687.7, 700.9, 704.6, 810.8, 854.0, 889.2, 912.4, 960.0, 966.9, 976.1, 989.4, 993.9, 1015.3, 1017.8, 1030.2, 1076.6, 1081.4, 1082.8, 1107.5, 1108.0, 1124.7, 1130.6, 1163.0, 1178.1, 1227.0, 1257.3, 1320.3, 1327.9, 1345.6, 1357.0, 1360.0, 1369.3, 1375.0, 1398.5, 1409.6, 1413.6, 1421.9, 1432.5, 1438.2, 1441.9, 1442.9, 1452.4, 1468.4, 1485.4, 1522.6, 1615.1, 2845.6, 2976.1, 2976.7, 2977.5, 2980.5, 2992.9, 3022.4, 3028.4, 3029.2, 3036.7, 3074.4, 3085.9, 3092.9, 3117.1, 3137.6, 3138.6, 3153.3 |
| tetraMF+ | 52.3, 58.5, 70.7, 81.4, 83.7, 88.6, 98.3, 115.8, 119.1, 124.4, 132.4, 198.3, 199.2, 204.4, 214.9, 228.9, 236.7, 276.7, 288.8, 293.0, 297.4, 301.9, 314.2, 317.5, 332.0, 348.3, 351.5, 365.3, 384.2, 405.2, 528.4, 549.4, 551.7, 559.1, 592.9, 619.3, 625.3, 627.2, 633.4, 636.1, 638.6, 641.3, 648.8, 660.0, 676.3, 683.6, 687.1, 691.2, 708.1, 819.5, 844.8, 929.6, 940.9, 961.8, 965.9, 984.4, 1001.6, 1031.5, 1058.7, 1061.2, 1066.9, 1091.1, 1098.8, 1109.3, 1137.8, 1150.0, 1168.4, 1176.8, 1239.6, 1294.6, 1328.7, 1337.2, 1359.5, 1367.7, 1379.5, 1393.6, 1396.8, 1412.6, 1413.4, 1424.0, 1433.2, 1440.2, 1457.8, 1460.4, 1478.9, 1552.9, 1628.1, 2926.5, 2950.1, 2955.3, 2983.1, 2986.6, 2993.8, 3016.8, 3056.2, 3068.9, 3078.6, 3088.9, 3091.3, 3096.6, 3133.5, 3186.6 |
| pentaMCP+ | 51.1, 55.1, 76.9, 84.6, 97.7, 100.3, 109.6, 120.3, 132.4, 145.2, 166.0, 192.9, 200.5, 206.8, 217.8, 226.3, 228.3, 233.3, 270.7, 285.8, 287.5, 297.9, 298.8, 313.4, 316.7, 340.3, 345.8, 355.2, 366.8, 393.8, 417.0, 523.4, 539.8, 552.2, 560.2, 567.4, 624.8, 628.1, 629.2, 633.0, 634.2, 640.1, 642.7, 650.8, 659.4, 682.2, 686.2, 691.6, 694.6, 817.2, 844.3, 910.0, 924.4, 941.5, 967.4, 990.4, 1009.2, 1030.2, 1044.1, 1061.7, 1077.4, 1082.1, 1091.2, 1103.6, 1129.1, 1132.1, 1153.9, 1155.6, 1168.9, 1193.1, 1227.0, 1262.2, 1309.3, 1311.0, 1319.5, 1339.2, 1373.1, 1377.1, 1379.0, 1387.0, 1392.5, 1410.3, 1424.2, 1430.0, 1442.5, 1448.1, 1455.4, 1459.6, 1462.0, 1496.9, 1550.6, 2869.3, 2900.9, 2901.9, 2934.6, 2995.0, 2996.1, 2998.2, 3005.3, 3027.9, 3069.1, 3071.1, 3088.7, 3089.0, 3095.7, 3097.7, 3107.3, 3111.0 |
| H-ZSM-5 co-adsorbates |  |
| hexaMB_CD_3_CN | 5.8, 47.2, 60.1, 76.9, 83.6, 92.2, 99.6, 103.7, 105.4, 112.5, 121.3, 126.4, 133.7, 144.3, 165.4, 183.0, 187.2, 191.7, 201.9, 211.1, 217.0, 221.7, 226.5, 244.6, 261.4, 263.8, 274.8, 288.4, 305.1, 330.4, 333.1, 340.0, 344.0, 348.5, 350.3, 356.0, 360.6, 365.4, 391.2, 408.6, 410.4, 448.2, 452.5, 462.2, 557.7, 561.0, 565.2, 570.4, 576.3, 602.6, 621.9, 628.9, 645.3, 647.5, 653.0, 656.6, 666.3, 678.7, 688.5, 700.6, 716.0, 721.2, 800.0, 812.9, 831.0, 832.5, 849.6, 878.6, 955.6, 971.6, 975.2, 980.4, 995.1, 996.2, 998.3, 1002.4, 1008.5, 1022.4, 1028.1, 1029.8, 1052.3, 1063.5, 1076.1, 1079.9, 1096.3, 1098.0, 1101.1, 1107.0, 1141.5, 1251.1, 1308.7, 1340.1, 1349.4, 1353.2, 1357.0, 1359.8, 1364.1, 1377.8, 1379.5, 1405.8, 1409.9, 1420.6, 1424.4, 1425.6, 1427.6, 1436.6, 1449.3, 1450.7, 1455.8, 1458.8, 1462.1, 1469.4, 1487.8, 1581.9, 1588.4, 2037.3, 2151.7, 2277.6, 2284.6, 2358.3, 2955.5, 2963.3, 2966.4, 2972.7, 2976.9, 2989.1, 3004.8, 3009.2, 3012.0, 3021.0, 3025.7, 3066.1, 3073.9, 3111.1, 3138.4, 3144.2, 3181.7, 3200.4 |
| pentaMB_CD_3_CN | 30.3, 43.0, 53.2, 75.6, 81.8, 83.8, 96.9, 100.2, 106.3, 109.0, 121.3, 140.7, 153.0, 157.9, 174.7, 197.9, 205.7, 212.4, 217.7, 224.7, 230.6, 253.0, 261.6, 265.7, 273.7, 278.2, 307.3, 309.2, 314.3, 329.1, 332.6, 342.9, 345.0, 347.5, 351.9, 362.6, 365.2, 387.5, 410.7, 455.5, 484.8, 522.7, 544.7, 557.0, 576.9, 591.7, 621.5, 632.1, 643.5, 648.1, 653.9, 655.6, 664.2, 681.2, 684.7, 690.8, 701.1, 715.4, 718.8, 818.2, 828.3, 831.6, 844.8, 848.8, 877.9, 898.9, 964.9, 982.2, 996.2, 999.0, 999.6, 1000.7, 1009.4, 1017.0, 1025.3, 1029.5, 1033.4, 1066.3, 1076.6, 1097.5, 1098.3, 1102.4, 1104.0, 1141.8, 1222.6, 1225.6, 1315.8, 1341.4, 1351.1, 1354.7, 1365.0, 1365.4, 1378.1, 1379.4, 1397.3, 1414.6, 1421.3, 1423.3, 1429.0, 1437.0, 1444.8, 1446.5, 1456.3, 1460.8, 1466.7, 1479.9, 1584.2, 1616.2, 2038.5, 2150.9, 2277.3, 2283.2, 2358.5, 2959.5, 2964.8, 2968.7, 2975.0, 2998.2, 3000.6, 3009.4, 3019.5, 3027.4, 3057.2, 3060.4, 3077.6, 3101.5, 3107.8, 3183.8, 3203.8 |
| tetraMF_CD_3_CN | 42.8, 49.8, 61.4, 73.3, 77.2, 81.2, 84.5, 96.5, 103.3, 113.5, 118.9, 121.8, 140.5, 147.6, 158.7, 164.9, 187.1, 204.3, 206.8, 214.8, 227.3, 235.3, 244.4, 259.4, 260.9, 274.0, 278.3, 296.4, 304.1, 305.6, 309.0, 327.7, 342.8, 343.9, 347.1, 361.7, 376.1, 404.9, 516.9, 555.3, 557.5, 569.5, 580.1, 620.7, 627.5, 634.8, 642.2, 648.7, 652.7, 654.0, 663.8, 682.2, 683.3, 691.6, 703.0, 716.9, 720.2, 799.9, 828.6, 830.7, 835.3, 847.9, 879.1, 905.4, 934.1, 952.6, 987.0, 998.6, 1001.5, 1003.7, 1017.5, 1020.0, 1030.4, 1071.2, 1086.8, 1096.8, 1100.3, 1102.4, 1106.6, 1143.2, 1155.6, 1222.0, 1318.4, 1333.7, 1360.2, 1368.2, 1374.5, 1381.7, 1390.9, 1407.4, 1416.2, 1423.4, 1426.1, 1427.2, 1433.9, 1437.7, 1441.3, 1456.5, 1611.8, 1655.2, 1666.3, 2014.4, 2148.7, 2273.6, 2281.9, 2360.7, 2949.2, 2956.8, 2961.8, 2978.4, 2997.0, 3007.6, 3008.4, 3024.3, 3034.1, 3040.5, 3089.7, 3101.8, 3103.6, 3252.9 |
| pentaMCP_CD_3_CN | 33.9, 49.5, 62.5, 64.5, 67.7, 79.4, 83.8, 88.5, 96.6, 100.7, 103.0, 107.4, 112.5, 132.2, 145.0, 155.5, 160.4, 177.0, 193.3, 207.0, 212.1, 221.5, 226.5, 238.2, 262.1, 272.5, 275.3, 277.7, 281.4, 306.2, 318.7, 329.8, 342.0, 346.7, 354.6, 363.0, 365.4, 382.8, 403.2, 495.8, 550.4, 555.2, 555.5, 591.8, 617.7, 633.8, 635.6, 645.5, 648.2, 652.1, 653.9, 666.9, 687.9, 696.1, 699.7, 710.8, 724.2, 788.8, 827.1, 834.2, 837.9, 842.8, 869.4, 931.9, 937.7, 959.2, 984.0, 987.5, 995.8, 1007.1, 1012.4, 1015.2, 1022.2, 1050.0, 1050.3, 1068.5, 1101.5, 1103.9, 1109.9, 1111.0, 1137.3, 1147.8, 1159.3, 1196.9, 1255.4, 1313.9, 1320.0, 1341.2, 1354.2, 1359.8, 1362.8, 1363.4, 1366.0, 1418.3, 1420.1, 1420.7, 1424.2, 1428.4, 1429.9, 1433.7, 1435.0, 1443.7, 1466.6, 1613.5, 1665.8, 2139.9, 2154.4, 2270.4, 2277.1, 2351.1, 2929.4, 2933.7, 2948.8, 2964.4, 2969.6, 2973.6, 2978.5, 2993.3, 2995.2, 3034.9, 3055.0, 3078.5, 3083.8, 3085.0, 3091.8, 3108.4 |
| hexaMB_pyridine | 44.0, 58.0, 62.0, 71.5, 74.1, 85.8, 86.2, 94.4, 101.7, 105.8, 112.5, 123.3, 133.5, 141.6, 151.0, 176.4, 192.1, 196.9, 203.2, 210.2, 211.0, 223.0, 228.9, 244.4, 268.7, 277.7, 282.0, 300.3, 308.5, 332.8, 339.8, 345.4, 355.3, 358.4, 362.7, 366.9, 393.3, 397.0, 405.2, 412.4, 420.7, 442.9, 451.0, 465.0, 545.8, 560.2, 563.7, 573.2, 580.7, 603.1, 607.1, 629.3, 632.4, 634.1, 634.7, 639.6, 642.3, 646.2, 651.4, 662.3, 672.1, 685.0, 690.9, 699.9, 720.7, 746.0, 799.3, 811.9, 860.2, 928.5, 951.1, 968.1, 971.0, 974.2, 978.6, 987.6, 994.6, 998.0, 1007.9, 1009.6, 1021.0, 1024.8, 1030.8, 1032.7, 1051.5, 1061.0, 1061.4, 1071.1, 1075.5, 1080.0, 1093.2, 1096.6, 1109.7, 1152.9, 1153.9, 1164.9, 1194.2, 1253.3, 1299.1, 1304.7, 1341.7, 1347.5, 1351.2, 1352.0, 1357.0, 1364.2, 1365.4, 1374.8, 1398.5, 1403.8, 1406.4, 1418.5, 1422.9, 1425.4, 1430.2, 1438.7, 1444.8, 1447.7, 1452.8, 1457.9, 1459.7, 1465.6, 1478.1, 1483.6, 1551.4, 1576.7, 1590.2, 1627.6, 1633.6, 2691.5, 2957.8, 2965.0, 2970.8, 2973.3, 2974.4, 2982.9, 3004.4, 3009.4, 3015.2, 3018.0, 3019.3, 3076.7, 3082.4, 3105.9, 3116.4, 3130.1, 3136.0, 3138.0, 3143.1, 3162.9, 3172.1, 3208.5, 3217.5 |
| pentaMB_pyridine | 39.2, 51.4, 63.3, 69.5, 83.8, 86.8, 91.8, 93.9, 111.6, 123.0, 134.2, 136.8, 149.5, 162.9, 188.8, 198.8, 203.7, 204.8, 213.0, 217.9, 227.0, 244.1, 265.5, 269.1, 269.4, 270.7, 304.5, 310.0, 316.9, 334.4, 338.5, 347.3, 349.1, 366.2, 375.4, 394.6, 399.9, 402.6, 420.9, 456.1, 485.6, 520.1, 545.9, 546.7, 573.6, 594.9, 607.8, 630.8, 632.2, 633.8, 636.2, 642.5, 643.6, 648.6, 652.1, 660.1, 672.8, 684.6, 686.3, 689.4, 700.1, 714.7, 743.5, 817.0, 845.7, 859.8, 897.1, 928.8, 963.0, 970.6, 983.2, 987.8, 996.6, 1000.2, 1006.9, 1009.0, 1015.0, 1024.5, 1029.9, 1031.0, 1031.6, 1061.3, 1066.4, 1071.4, 1076.6, 1091.1, 1099.9, 1106.6, 1150.3, 1153.8, 1163.9, 1193.4, 1223.3, 1225.7, 1298.7, 1314.9, 1341.1, 1350.2, 1350.9, 1354.9, 1365.5, 1367.5, 1375.5, 1395.7, 1399.5, 1412.5, 1422.0, 1424.2, 1427.5, 1436.9, 1444.3, 1450.9, 1455.9, 1458.7, 1465.3, 1478.3, 1478.6, 1550.9, 1581.7, 1614.2, 1627.2, 1634.5, 2693.2, 2962.0, 2965.4, 2969.3, 2974.2, 2990.7, 3006.7, 3013.5, 3018.2, 3022.2, 3052.0, 3069.4, 3085.1, 3105.0, 3110.0, 3122.1, 3131.1, 3144.1, 3162.6, 3171.4, 3204.6, 3212.6 |
| tetraMF_pyridine | 38.6, 54.9, 60.8, 65.4, 71.3, 74.7, 85.1, 96.1, 99.9, 104.0, 113.7, 128.7, 132.6, 150.0, 156.2, 158.7, 162.7, 173.3, 189.7, 205.9, 212.2, 219.4, 243.0, 262.4, 276.6, 285.7, 286.4, 295.1, 302.0, 305.0, 324.2, 332.7, 356.1, 360.1, 384.8, 387.5, 403.1, 413.6, 528.6, 543.2, 553.8, 563.4, 599.4, 605.2, 631.8, 632.6, 632.9, 634.4, 636.9, 638.0, 639.7, 646.8, 649.7, 657.6, 675.2, 681.5, 685.8, 691.6, 699.0, 741.4, 742.9, 811.4, 828.8, 861.0, 909.3, 931.8, 939.9, 953.4, 973.9, 995.0, 1000.6, 1007.5, 1008.5, 1012.5, 1020.9, 1028.8, 1041.2, 1059.9, 1070.4, 1071.1, 1087.8, 1105.3, 1105.6, 1113.4, 1131.1, 1153.8, 1164.4, 1166.3, 1194.9, 1215.1, 1301.8, 1315.9, 1335.5, 1350.4, 1357.9, 1362.9, 1371.4, 1384.6, 1398.0, 1415.9, 1418.6, 1425.1, 1427.1, 1431.4, 1437.3, 1438.5, 1444.8, 1454.6, 1477.7, 1552.4, 1605.9, 1630.5, 1632.0, 1651.9, 1687.2, 2752.8, 2940.8, 2941.4, 2950.4, 2977.8, 2983.2, 2984.2, 2998.1, 3047.5, 3056.8, 3072.8, 3087.4, 3089.2, 3113.0, 3117.7, 3130.2, 3143.7, 3152.9, 3162.0, 3206.1 |
| pentaMCP_pyridine | 45.4, 49.7, 63.9, 67.1, 74.1, 80.6, 86.8, 91.3, 100.9, 108.8, 116.0, 118.3, 121.0, 130.8, 155.2, 162.5, 169.4, 178.9, 195.7, 199.9, 218.0, 222.4, 239.8, 250.6, 274.8, 278.7, 282.9, 285.8, 303.1, 317.6, 331.5, 347.0, 352.6, 362.4, 373.6, 375.2, 387.4, 394.7, 403.8, 502.2, 550.3, 552.7, 555.6, 588.5, 602.4, 628.2, 630.0, 633.1, 635.3, 636.8, 638.9, 640.7, 651.4, 658.6, 659.0, 671.7, 680.1, 689.4, 692.9, 706.8, 744.3, 792.7, 834.3, 860.2, 905.5, 933.5, 939.5, 956.4, 973.3, 977.3, 983.6, 994.0, 1013.0, 1014.2, 1022.4, 1028.3, 1032.7, 1044.1, 1048.6, 1057.0, 1062.4, 1068.8, 1069.9, 1079.4, 1105.0, 1115.4, 1141.0, 1154.5, 1156.9, 1163.8, 1189.9, 1197.2, 1256.4, 1291.6, 1316.7, 1322.5, 1349.2, 1351.8, 1356.9, 1359.9, 1366.7, 1369.1, 1393.9, 1416.7, 1421.1, 1425.7, 1428.2, 1428.4, 1431.8, 1432.7, 1443.6, 1447.3, 1463.8, 1478.3, 1544.8, 1608.9, 1620.8, 1635.8, 1660.4, 2929.2, 2938.2, 2946.4, 2972.4, 2974.3, 2975.2, 2975.3, 2984.9, 2987.1, 3000.6, 3041.1, 3062.0, 3075.7, 3089.9, 3095.7, 3098.2, 3102.2, 3129.0, 3145.6, 3148.8, 3161.5, 3175.5 |

**Table S6**. Proton affinity (PA) values of various bases.

| **Entry** | **Name** | **PA (kJ/mol)** | **Conjugate acid** | **Ref.** |
| --- | --- | --- | --- | --- |
| CH_3_OH | Methanol | 754.3 | CH_3_OH_2_^+^ | ^13^ |
| CD_3_CN | Acetonitrile-D_3_ | 779.5 | CD_3_CNH^+^ | ^14^ |
| C_9_H_12_ | 1,3,5-trimethylbenzene | 836.2 | C_9_H_13_^+^ | ^13^ |
| NH_3_ | Ammonia | 853.6 | NH_4_^+^ | ^13^ |
| C_12_H_18_ | Hexamethylbenzene | 872.8 | C_12_H_19_^+^ | ^13^ |
| C_7_H_10_ | 1,3-dimethylcyclopentadiene | 902.1 | C_7_H_11_^+^ | ^15^ |
| C_5_H_5_N | Pyridine | 930.0 | C_5_H_5_NH^+^ | ^13^ |
| Si-O^-^-Al | Zeolitic bridging oxygen | 1139-1204 | Si-(OH)-Al | ^16^ |

**References**

(1) Maggiulli, L.; Sushkevich, V. L.; Kröcher, O.; van Bokhoven, J. A.; Ferri, D. Correlating the Nature of Carbenium Ions in Zeolites to the Product Distribution in the Methanol-to-Olefins Process. *ACS Catal.* **2024**, 11477-11489. DOI: 10.1021/acscatal.4c03185.

(2) Wichterlová, B.; Tvarůžková, Z.; Sobalık, Z.; Sarv, P. Determination and Properties of Acid Sites in H-Ferrierite: A Comparison of Ferrierite and Mfi Structures. *Microporous Mesoporous Mater.* **1998**, *24*, 223-233.

(3) Plessow, P. N.; Studt, F. Theoretical Insights into the Effect of the Framework on the Initiation Mechanism of the Mto Process. *Catal. Lett.* **2018**, *148*, 1246-1253.

(4) Larsen, A. H.; Mortensen, J. J.; Blomqvist, J.; Castelli, I. E.; Christensen, R.; Dułak, M.; Friis, J.; Groves, M. N.; Hammer, B.; Hargus, C. The Atomic Simulation Environment—a Python Library for Working with Atoms. *J. Phys.: Condens. Matter* **2017**, *29*, 273002.

(5) Perdew, J. P.; Burke, K.; Ernzerhof, M. Generalized Gradient Approximation Made Simple. *Phys. Rev. Lett.* **1996**, *77*, 3865.

(6) Grimme, S.; Antony, J.; Ehrlich, S.; Krieg, H. A Consistent and Accurate Ab Initio Parametrization of Density Functional Dispersion Correction (Dft-D) for the 94 Elements H-Pu. *The Journal of chemical physics* **2010**, *132*.

(7) Kresse, G.; Joubert, D. From Ultrasoft Pseudopotentials to the Projector Augmented-Wave Method. *Physical review b* **1999**, *59*, 1758.

(8) Enss, A. E.; Plessow, P. N.; Studt, F. Theoretical Investigation of the Paring Mechanism of the Mto Process in Different Zeolites. *J. Catal.* **2024**, *432*, 115363.

(9) Brogaard, R. Y.; Wang, C.-M.; Studt, F. Methanol–Alkene Reactions in Zeotype Acid Catalysts: Insights from a Descriptor-Based Approach and Microkinetic Modeling. *ACS Catal.* **2014**, *4*, 4504-4509.

(10) Brogaard, R. Y.; Henry, R.; Schuurman, Y.; Medford, A. J.; Moses, P. G.; Beato, P.; Svelle, S.; Nørskov, J. K.; Olsbye, U. Methanol-to-Hydrocarbons Conversion: The Alkene Methylation Pathway. *J. Catal.* **2014**, *314*, 159-169.

(11) Baerlocher, C.; McCusker, L. B. Database of Zeolite Structures. <http://www.iza-structure.org/databases/>.

(12) Rouquerol, J.; Llewellyn, P.; Rouquerol, F. Is the Bet Equation Applicable to Microporous Adsorbents. *Stud. Surf. Sci. Catal* **2007**, *160*, 49-56.

(13) Hunter, E. P.; Lias, S. G. Evaluated Gas Phase Basicities and Proton Affinities of Molecules: An Update. *J. Phys. Chem. Ref. Data* **1998**, *27*, 413-656.

(14) Williams, T. I.; Denault, J. W.; Cooks, R. G. Proton Affinity of Deuterated Acetonitrile Estimated by the Kinetic Method with Full Entropy Analysis. *Int. J. Mass spectrom.* **2001**, *210*, 133-146.

(15) Song, W.; Nicholas, J. B.; Haw, J. F. Acid− Base Chemistry of a Carbenium Ion in a Zeolite under Equilibrium Conditions: Verification of a Theoretical Explanation of Carbenium Ion Stability. *Journal of the American Chemical Society* **2001**, *123*, 121-129.

(16) Kubelková, L.; Beran, S.; Lercher, J. A. Determination of Proton Affinity of Zeolites and Zeolite-Like Solids by Low-Temperature Adsorption of Carbon Monoxide. *Zeolites* **1989**, *9*, 539-543.
